# Elucidating the fate of nanoparticles among key cell components of the tumor microenvironment for promoting cancer nanotechnology

**DOI:** 10.1186/s12645-020-00064-6

**Published:** 2020-08-18

**Authors:** Kyle Bromma, Aaron Bannister, Antonia Kowalewski, Leah Cicon, Devika B. Chithrani

**Affiliations:** 1 Department of Physics and Astronomy, University of Victoria, Victoria, BC Canada; 2 Department of Physics, Simon Fraser University, Burnaby, BC Canada; 3 Centre for Advanced Materials and Related Technologies (CAMTEC), Victoria, BC Canada; 4 Centre for Biomedical Research, University of Victoria, Victoria, BC Canada

**Keywords:** Gold nanoparticles, Cancer cells, Normal fibroblast cells, Cancer-associated fibroblast cells, Uptake, Retention, Microtubules, Toxicity

## Abstract

Successful integration of nanotechnology into the current paradigm of cancer therapy requires proper understanding of the interface between nanoparticles (NPs) and cancer cells, as well as other key components within the tumor microenvironment (TME), such as normal fibroblasts (FBs) and cancer-associated FBs (CAFs). So far, much focus has been on cancer cells, but FBs and CAFs also play a critical role: FBs suppress the tumor growth while CAFs promote it. It is not yet known how NPs interact with FBs and CAFs compared to cancer cells. Hence, our goal was to elucidate the extent of NP uptake, retention, and toxicity in cancer cells, FBs, and CAFs to further understand the fate of NPs in a real tumor-like environment. The outcome of this would guide designing of NP-based delivery systems to fully exploit the TME for a better therapeutic outcome. We used gold nanoparticles as our model NP system due to their numerous applications in cancer therapy, including radiotherapy and chemotherapy. A cervical cancer cell line, HeLa, and a triple-negative breast cancer cell line, MDA-MB-231 were chosen as cancer cell lines. For this study, a clinically feasible 0.2 nM concentration of GNPs was employed. According to our results, the cancer cells and CAFs had over 25- and 10-fold higher NP uptake per unit cell volume compared to FBs, respectively. Further, the cancer cells and CAFs had over 30% higher NP retention compared to FBs. There was no observed significant toxicity due to GNPs in all the cell lines studied. Higher uptake and retention of NPs in cancer cells and CAFs *vs* FBs is very important in promoting NP-based applications in cancer therapy. Our results show potential in modulating uptake and retention of GNPs among key components of TME, in an effort to develop NP-based strategies to suppress the tumor growth. An ideal NP-based platform would eradicate tumor cells, protect FBs, and deactivate CAFs. Therefore, this study lays a road map to exploit the TME for the advancement of “smart” nanomedicines that would constitute the next generation of cancer therapeutics.
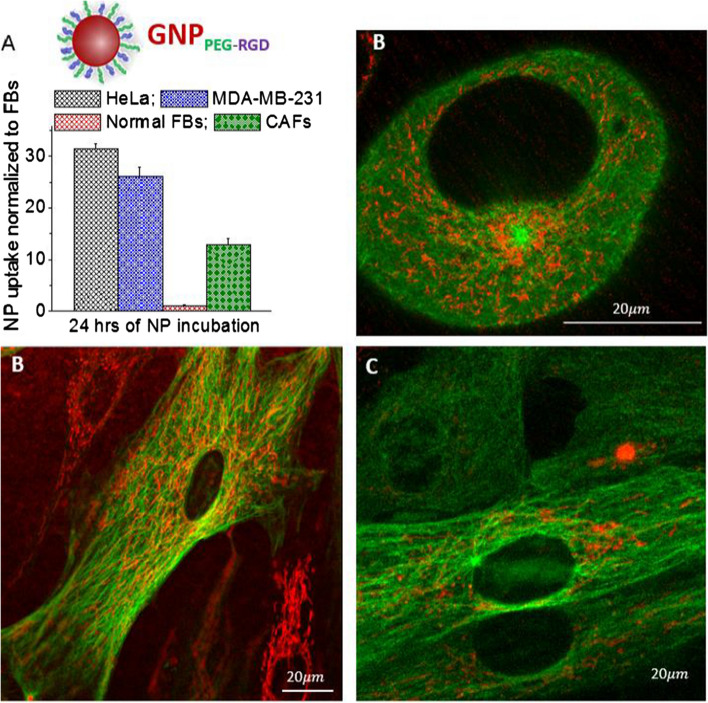

## Background

**Fig. 1:**
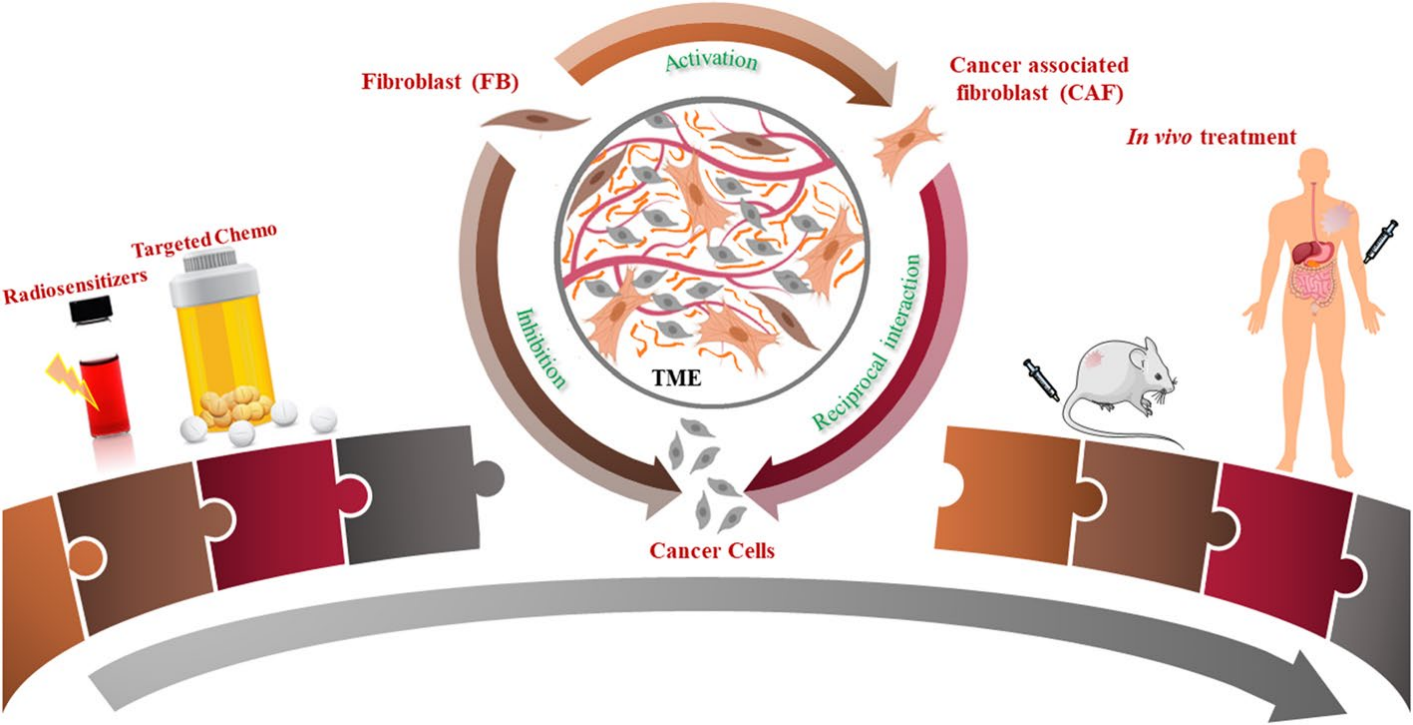
Nanoparticles are being widely explored in radiotherapy and chemotherapy (left most). The proper understanding of the interface between nanotechnology and tumor microenvironment (TME) involves elucidating the behavior of nanoparticles not only in cancer cells but also in other key components of TME, such as cancer-associated fibroblasts (CAFs), and normal fibroblasts (FBs) (middle). The information gathered from this study will play a significant role in advancing “smart” nano-based medicines into future clinical trials after testing them successfully in vivo (right most)

According to the global cancer observatory (GLOBOCAN), in 2018, there were 18.1 million new cases of cancer worldwide, and 9.9 million cancer deaths (Bray et al. 2018). Most cancer therapies are currently limited to surgery, radiotherapy (RT), and chemotherapy (CT). In RT and CT, the maximum tolerated dose is being utilized to treat patients. Innovative approaches are essential to address one of the main issues in both RT and CT: normal tissue toxicity. Nanoparticle (NP)-based packages provide a platform to deliver targeted therapeutics, offering the means to further improve CT through controlled delivery of chemotherapeutics to tumor cells while local RT dose can be enhanced by targeting NP-based radiosensitizers to tumors. Most nanotechnology-based research has so far mainly focused on cancer cells, and not on other key cellular components within the tumor microenvironment (TME) (Miao and Huang 2015). As illustrated in thematic Fig. 1, the goal of this study is to elucidate the fate of NPs within key interrelated cellular components of the TME, which includes cancer cells, fibroblasts (FBs), and cancer-associated fibroblasts (CAFs), in order to fully exploit the promise of cancer nanomedicine.

The progression of a tumor depends on the dynamic interactions between tumor cells and their surrounding stroma within the TME (Alkasalias et al. 2018). The stroma includes the extra cellular matrix (ECM), the basement membrane, local immune cells, vasculature, and normal FBs. FBs, as the building blocks of connective tissues, are key components of the TME. Interactions between tumor cells and the surrounding FBs serve an important role in cancer proliferation. It has been suggested that FBs inhibit cancer cell proliferation and metastasis (Alkasalias et al. 2018). However, FBs present within a TME can be recruited by the cancer, turning them into CAFs to promote the tumor growth. CAFs are the most abundant cell type in the tumor stroma and are actively involved in tumor progression and invasion (Wang et al. 2017). Hence, in addition to tumor cells, CAFs and FBs are the most prominent cell types in the tumor stroma that require attention, in order to build successful NP-based therapeutic strategies to eradicate cancer (Anderberg and Pietras 2009). The potential of NP-based platforms in both RT and CT has been focused mainly on cancer cells; however, it is not yet known how NPs interact with other key components of TME such as FBs and CAFs. We used two tumor cell models for this study: HeLa, a cervical cancer cell line, and MDA-MB-231, a triple-negative breast cancer cell line. In order to study the fate of NPs across these stromal cells, we chose GNPs as our model NP system.

Among other NP systems, we used GNPs as a model NP system for this study due to their promising results in several practiced clinical applications including RT and CT as described in Fig. 1 (left most) (Chithrani et al. 2010; González-López et al. 2020; Khoo et al. 2017; Paciotti et al. 2016). Biocompatibility of GNPs and their ability to act as a vector for targeted drug delivery to the tumor were demonstrated successfully in a phase I clinical trial (Libutti et al. 2010). A systemic administration of the GNP–drug complex resulted in a delivery of drug doses that were previously shown to be toxic (Libutti et al. 2010). In RT, GNPs have been successfully tested as a promising radiosensitizer (Antosh et al. 2015; Bromma et al. 2019; Chithrani et al. 2010; Wolfe et al. 2015). The presence of GNPs during RT results in a higher cross section to low-energy photons, producing low-energy electrons and free radicals that could damage tumor cells (Carter et al. 2007; McMahon et al. 2011; Zheng and Sanche 2013). Using clinically relevant higher energy (mega electron volt) photon beams, many research groups have demonstrated the GNP-mediated dose enhancement at clinically feasible NP concentrations (Chithrani et al. 2010; Wolfe et al. 2015). In addition, gold-based NPs are also being explored in imaging, photothermal therapy, and as well as a tool for releasing drugs remotely (Ali et al. 2017; Chanda et al. 2010; Goodman et al. 2017). Therefore, the potential of GNPs in many cancer nanotechnology-based applications prompted its use as our model NP system in this study. The next important step was to decide on the size and surface properties of GNPs.

The size and surface properties of the NPs could influence their interaction at the individual cell level as well as within the TME. In monolayer cell cultures, the absence of the ECM does not affect transport of NPs compared to tissue-like structures where the ECM can act as a NP transport barrier. Among the size range of 10–100 nm, NPs of diameter 50 nm have shown the highest uptake in monolayer cell cultures (Chithrani et al. 2006; Gao et al. 2005). However, both the size of NPs and the presence of ECM are expected to play a significant role in their penetration and uptake in tissue-like (three dimensional in vitro) models. As expected, studies have shown that smaller NPs penetrate better through tissue compared to NPs of diameter 50 nm which was the optimum size in monolayer cell cultures (Yohan et al. 2016). Since smaller NPs have a higher probability of penetrating through the ECM once they leave the leaky vasculature present in tumors, increasing the uptake of those smaller NPs to be similar to that of 50-nm diameter ones is essential (Yang et al. 2018a). It has been shown that adding a peptide containing integrin-binding domain, RGD, could significantly improve the uptake of smaller NPs (Cruje 2015; Kim et al. 2011a; Yang et al. 2014; Yang et al. 2018b; Zhang et al. 2016). However, addition of RGD peptide requires stabilization of NPs to avoid aggregation. While the most used stabilization agent is pentapeptide, we used polyethylene glycol (PEG) molecules instead since an RGD/PEG combination would start bridging the gap between in vitro and in vivo, where stability and improved uptake is crucial. This will allow for translation of this work to future in vivo studies followed by clinical trials.

Our study aims at understanding of the differential uptake, distribution, retention, and toxicity of GNPs not only in cancer cells, but also in other two interrelated key cell types, FBs and CAFs in TME (Fig. 1; middle). The outcome of this study will promote designing of smart nanomaterials to yield optimum results in a real TME which would accelerate nano-based therapeutics in animal models followed by clinical translation as laid out in schematic Fig. 1 (right most). An ideal NP-based platform would eradicate tumor cells, protect FBs, and deactivate CAFs.

## Results and discussion

### Characterization of GNP complexes and determining their cellular uptake across key cellular components of the tumor microenvironment

**Fig. 2:**
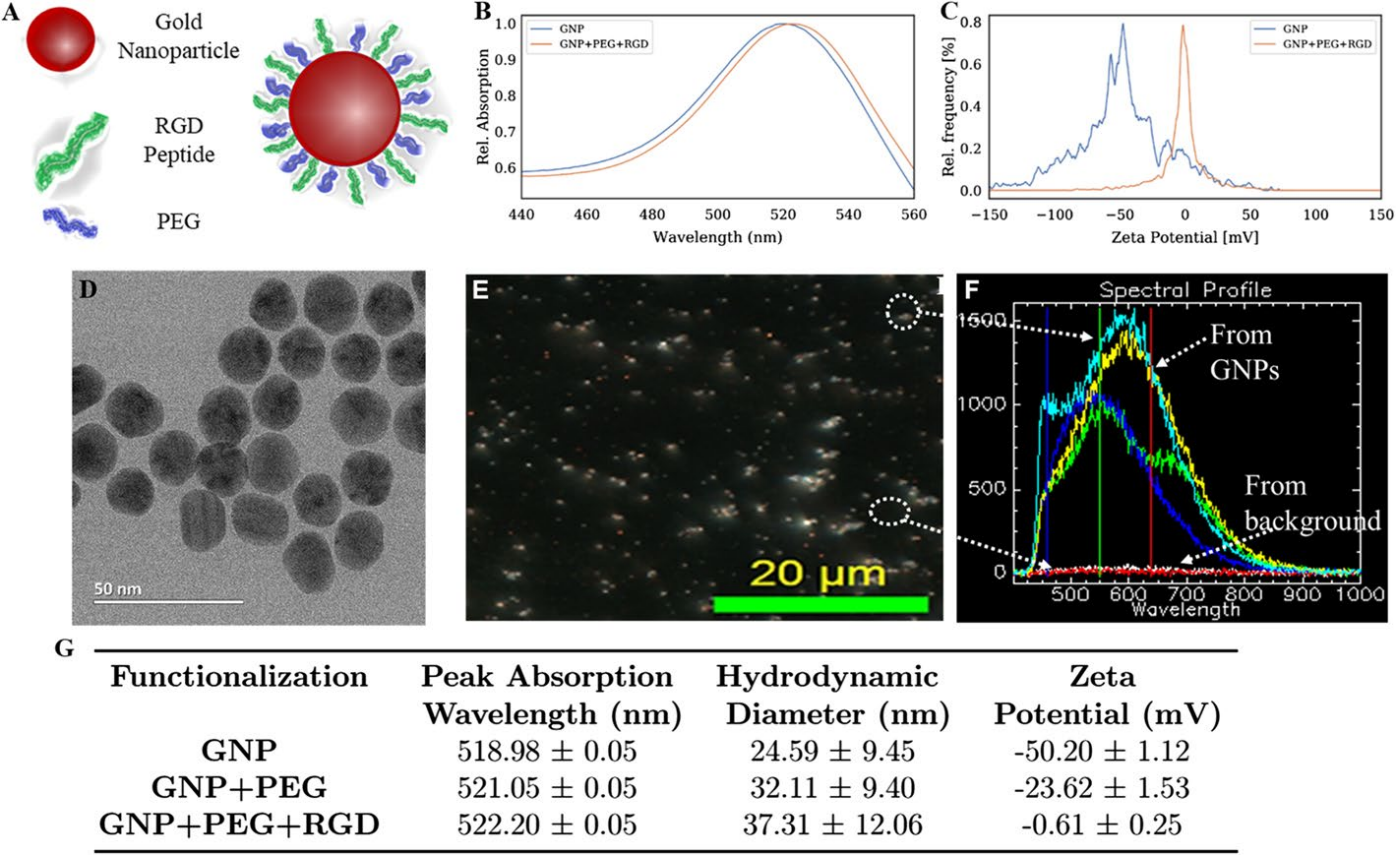
Characterization of gold nanoparticles (GNPs) that were used as the model NP system for this study. **a** Schematic diagram of a GNP functionalized with a peptide containing integrin-binding domain, RGD (referred to as RGD peptide) and polyethylene glycol (PEG). This GNP complex is referred to as GNP_PEG-RGD_. **b** Transmission electron microscopy (TEM) image of GNPs with measured core diameter of ~ 15 nm. **c, d** Darkfield image and spectral profile of GNPs, respectively. **e, f** UV–visible absorption spectra and $\zeta$-potential measurements of as-made GNPs and GNP_PEG–RGD_. (**g**) Summary of characterization data for as-made GNPs, GNP_PEG_, and GNP_PEG-RGD_. The measurements were done using three different samples (n = 3) and error represents standard deviation

To study the uptake cross-section among key cellular components within the TME, we used smaller sized GNPs of diameter ~ 15 nm functionalized with both PEG and RGD peptide (Fig. 2a). The rationale behind choosing this particular size and functional molecules was given in the introduction section. Based on our transmission electron microscopy (TEM) imaging, the average core diameter of synthesized GNPs was 16.5 ±  3.6 nm (Fig. 2b). In addition, both dark-field and hyperspectral imaging technology were employed to visualize GNPs. The dark-field image of GNPs used for the study and their corresponding reflectance spectra are given in Fig. 2c, d, respectively. The spectra with higher intensity represent data collected from GNPs while the flat spectra represent the signal from the background areas where there were no GNPs. The peak wavelength of UV–visible absorption spectrum of bare GNPs was 518 nm and it is aligned with the peak wavelength for 15–17 nm GNPs (Fig. 2e) (Haiss et al. 2007). There was only a slight red shift of the surface plasmon absorption peak wavelength for RGD/PEG modified GNPs (GNP_PEG-RGD_) since both RGD-peptide and PEG molecules were considerably smaller. For example, the molecular weight of RGD-peptide and PEG were 1760 and 2000 Da, respectively. However, the addition of PEG and RGD peptide resulted in a replacement of negatively charged citrate molecules which led to a significant change in the surface charge (Fig. 2f). The change in the hydrodynamic diameter was also measured and the results are listed in Fig. 2g. GNP_PEG–RGD_ complex was used for this study to determine the differential uptake of GNPs among HeLa and MDA-MB-231 (cancer cell lines), FBs (normal cell line), and CAFs as discussed in the next section.

**Fig. 3:**
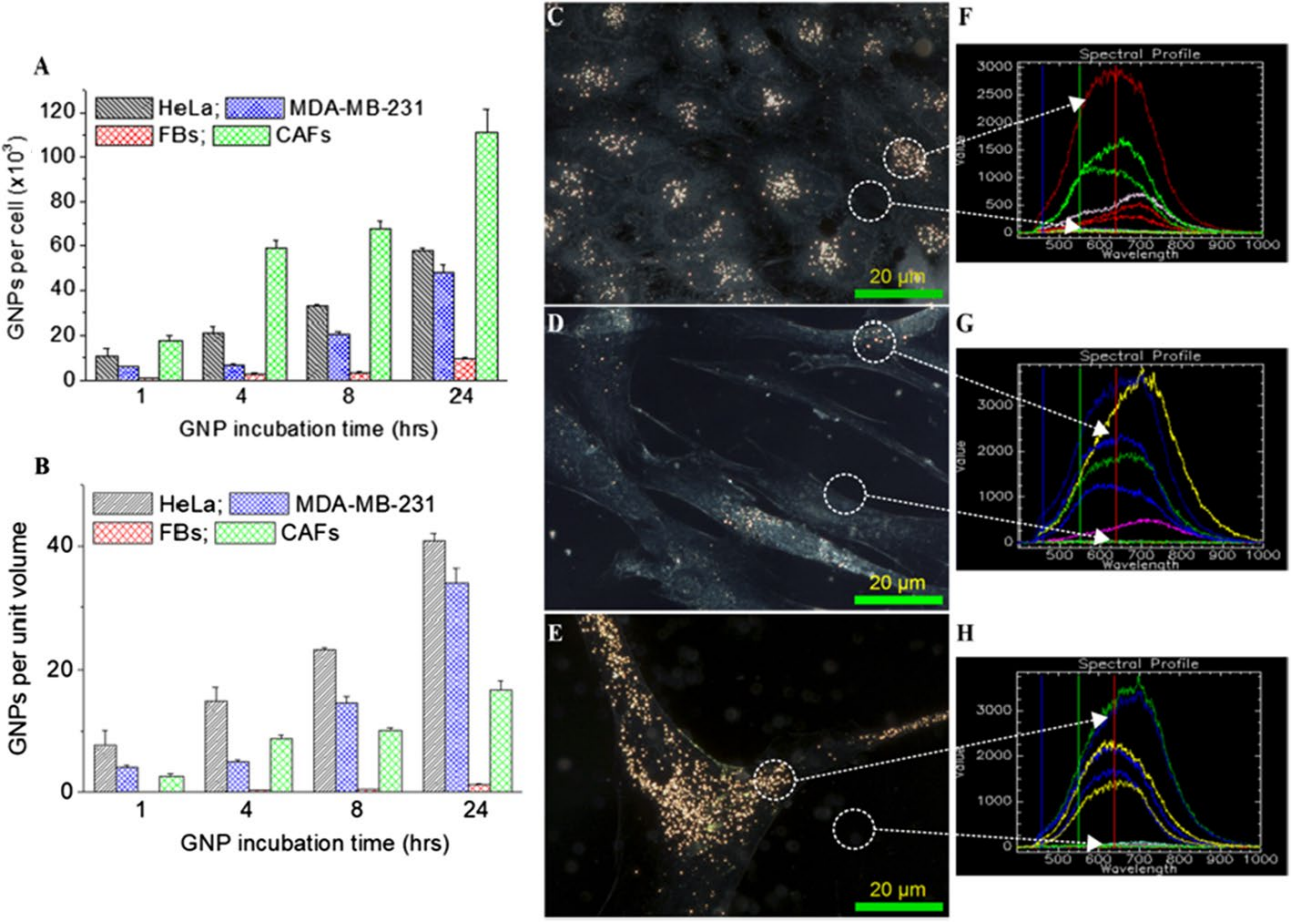
Quantitative and qualitative analysis of cellular uptake of GNP_PEG-RGD_. **a, b** Quantification of NP uptake per cell and per unit volume of the cell. **c–e** Darkfield images of HeLa, FBs, and CAFs, respectively. **f–h** Spectra collected from GNP clusters localized within the cells and from background in HeLa, FBs, and CAFs, respectively. Experiments were repeated three times (n = 3) and the data presented is the average. The error bars represent standard error. Scale bar = 20 μm

The majority of NPs are taken up by cells through a receptor mediated endocytosis (RME) process (Chithrani et al. 2006; Zhang et al. 2015). Once internalized, NPs get trapped in endosomes followed by fusion with lysosomes for further processing around the perinuclear region. Most of the receptors are recycled back to the cellular membrane while vesicles containing processed NPs head towards cell periphery for excretion (Chithrani and Chan 2007; Chithrani 2010). The process of cellular uptake of GNPs is dynamic and the number of GNPs present per cell for HeLa, MDA-MB-231, FBs, and CAFs within a 24 h incubation time period is plotted in Fig. 3a. Our GNP uptake experiments were carried out at 0.2 nM, since such concentrations are more relevant in vivo and the outcome of this study could be a useful resource to extrapolate meaningful conclusions for future clinical applications (Wolfe et al. 2015; Yang et al. 2018a). After an incubation time period of 24 h, CAFs and cancer cells (both HeLa and MDA-MB-231) had a much higher NP uptake both per cell and per unit volume in comparison to FBs (Fig. 3a, b). According to Fig. 3a, GNP uptake per cell in cancer cells and CAFs was ~ 6- and ~ 12-fold higher compared to FBs, respectively (Fig. 3a). We also looked at the presence of NPs per unit volume due to the significant differences in size and morphology among these cells as seen in Fig. 3c–e. According to Fig. 3b, cancer cells and CAFs had a ~ 25- and ~ 10-fold higher uptake per unit cell volume compared to FBs, respectively. This result is very promising considering one of the major concerns in NP-based therapeutics is the normal tissue toxicity. The FBs are also present within the TME where they can exert diverse suppressive functions against cancer initiation and metastatic behavior (Alkasalias et al. 2018). Having a significantly lower number of NPs in normal FBs would produce less damage and is very encouraging. The presence of significantly higher number of GNPs in cancer-associated cells such as HeLa, MDA-MB-231, and CAFs is also very promising in both RT and CT applications, since it would result in a higher RT dose and a more optimum delivery of drugs, causing the necessary damage to eradicate the tumor.

The intracellular distribution of GNP complex (GNP_PEG-RGD_) within HeLa, FBs, and CAFs was captured using dark field microscopy as shown in Fig. 3c–e, respectively. The imaging data corresponding to MDA-MB-231 cell line are given in the Additional file 1: Section S1. In this study, we first synthesized GNPs (as-made GNPs), secondly added PEG onto to as-made GNPs (GNP_PEG_), and finally added RGD peptide onto GNP_PEG_ complex (GNP_PEG-RGD_). We also followed the differences in intracellular distribution corresponding to two intermediate NP complexes, such as as-made GNPs and GNP_PEG_ using darkfield imaging (see the Additional file 1: Section S2). The results are consistent with previously published work for MDA-MB-231 cell line (Cruje et al. 2015). For example, the addition of PEG onto as-made GNPs resulted in a significant decrease in NP uptake. However, the addition of RGD peptides onto the GNP_PEG_ complex resulted in a significant increase in NP uptake.

The presence of higher number of GNPs in cancer cells and CAFs compared to FBs was apparent from these images (Fig. 3c–e). The reflectance spectra collected from NP clusters and background are displayed in Fig. 3f–h. Based on both quantitative and qualitative data, it is evident that cancer cells (HeLa and MDA-MB-231; see Additional file 1 for MDA-MB-231) and CAFs can be populated with significantly larger densities of GNP compared to normal FBs. As described previously, CAFs are the most abundant cells of the tumor stroma, where they substantially influence tumor growth through control of the surrounding TME (Mardhian et al. 2018; Mertens et al. 2013). As a result of the larger uptake of the GNPs in CAFs, researchers have the opportunity to build nano-strategies to eradicate not just the cancer cells, but also the supporting cells, to fully eliminate the tumor (Truffi et al. 2019).

### Intracellular distribution of NPs

**Fig. 4:**
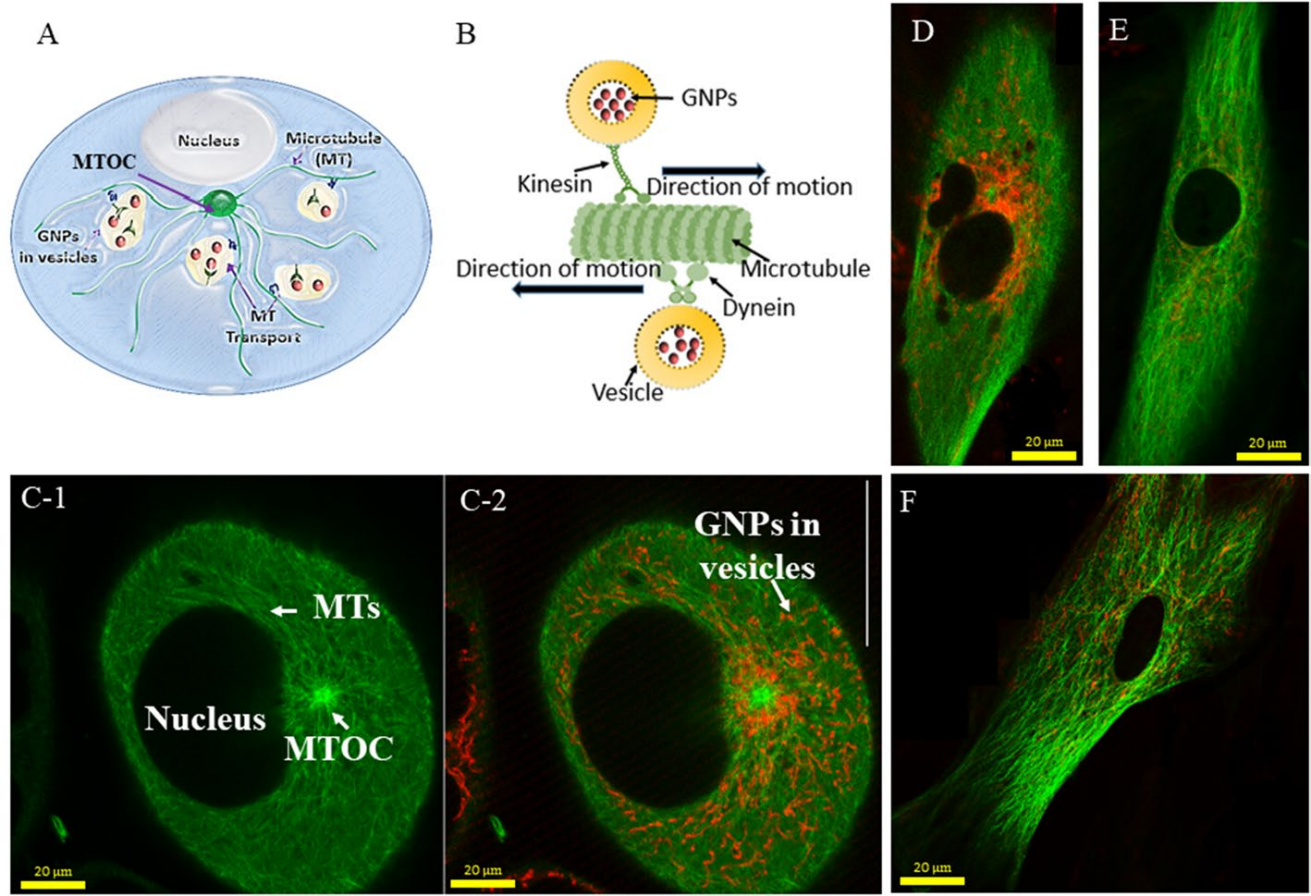
Microtubule (MT) network and distribution of NPs within the cell. **a** Schematic diagram of a cell illustrating the transport of vesicles along the MTs within the cell. **b** Schematic explaining the directional transport of vesicles containing GNPs along the MTs. **c** MT network (1) of a HeLa cell and the merged image (2) of the MT network and distribution of vesicles containing GNPs. MTs and vesicles containing GNPs are labeled in green and red, respectively. **d, e** MT network and GNP distribution in FBs and CAFs, respectively

The microtubules (MTs) in the cytoskeleton of cells play an important role in transporting these NP complexes within cells as illustrated in Fig. 4 (Gradishar 2012; Granger et al. 2014; Paoletti et al. 1997). These MTs are arranged radially, nucleating from a microtubule organizing center (MTOC) near the nucleus and extending towards the cell membrane (Fig. 4a). Motor proteins such as dynein and kinesin play a significant role in trafficking organelles and vesicles containing NP clusters within the cell (Kulić et al. 2008). For example, kinesin and dynein move vesicles containing cargo such as NPs in opposite directions along microtubules as shown in Fig. 4b. A confocal image slice across the nucleus of a HeLa cell is presented in Fig. 4c-1 where the MTOC and MT network (labeled in green) are clearly seen. An image taken at the depth of the nucleus ensures that the imaged GNPs, as well as other properties, are contained within the cell and not adhered to the surface. The merged image of vesicles containing GNPs (marked in red) and MT network is displayed in Fig. 4c-2. The images in Fig. 4d–f show the MT network and vesicles containing NPs within MDA-MB-231 Cells, FBs and CAFs, respectively. It is evident from these images that cancer cells (HeLa and MDA-MB-231) and CAFs had a significantly higher presence of GNPs as compared to normal cells, i.e., FBs, consistent with our quantitative and qualitative data in Fig. 3. It is also clear that NPs were localized only within the cytoplasm and not in the nucleus, as expected. Figure 3 has images of individual cells and Additional file 1: S3–S6 were added to include additional images for further illustrations. A recent study has demonstrated how this MT network can be manipulated using a taxane-based anticancer drug, docetaxel, to redistribute GNPs closer to the nucleus for optimum outcome in RT (Bannister et al. 2019). The use of docetaxel as a novel strategy in the future could significantly improve RT and CT, since both cancer cells and CAFs take up a significantly higher number of GNPs compared to normal FBs, in accordance with our results.

### Processing and retention of NPs

**Fig. 5:**
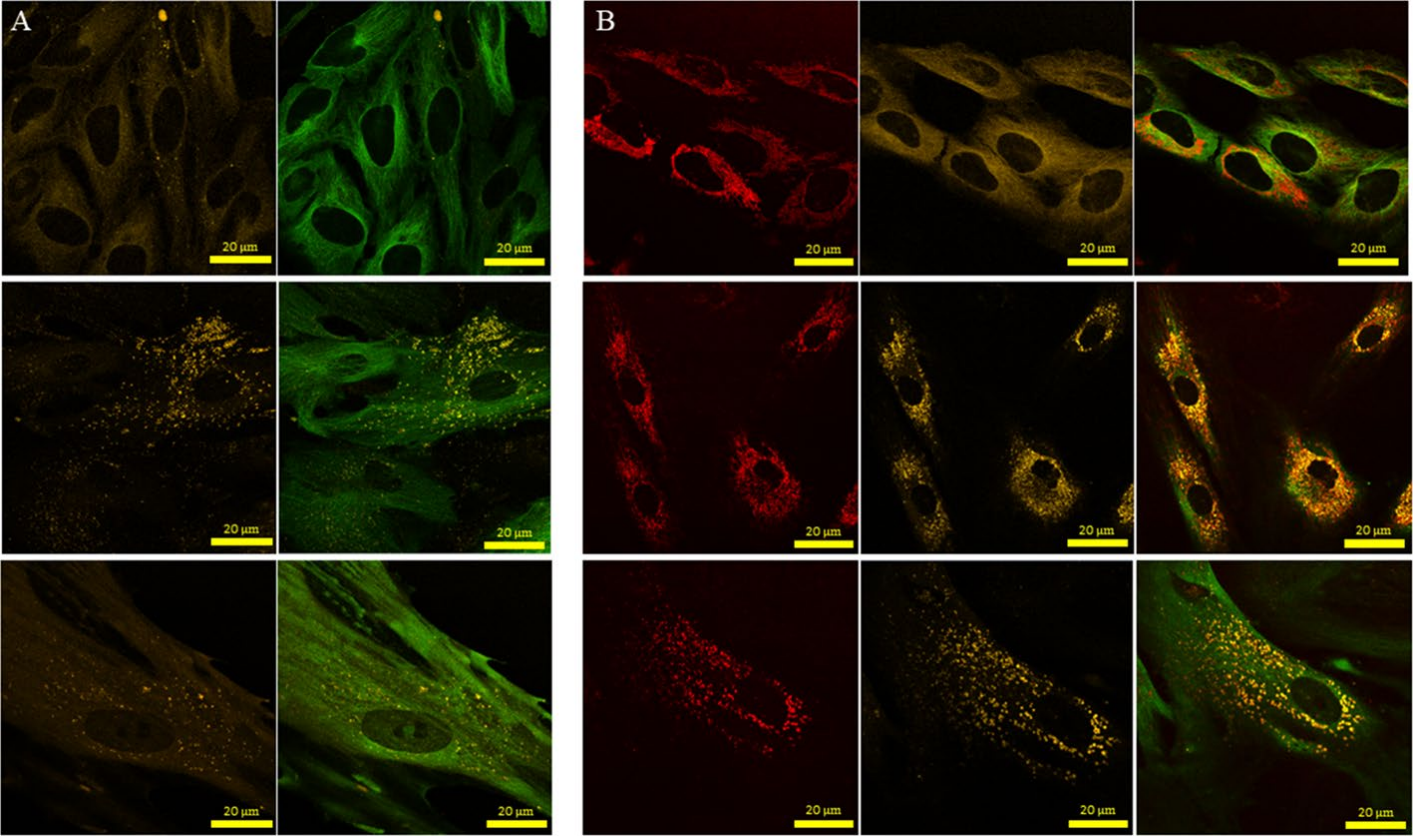
Endolysosomal distribution in **a** control cells and in **b** cells treated with GNPs. Images in first, second and third rows correspond to HeLa cells, FBs, and CAFs, respectively. In panel **a**, the first column shows the distribution of lysosomes while the second column shows the merged image of lysosomes plus MTs. In panel **b**, the first, second, and third columns show the distribution of NPs, lysosomes, and merged image of lysosomes, MTs, and NPs, respectively

**Fig. 6:**
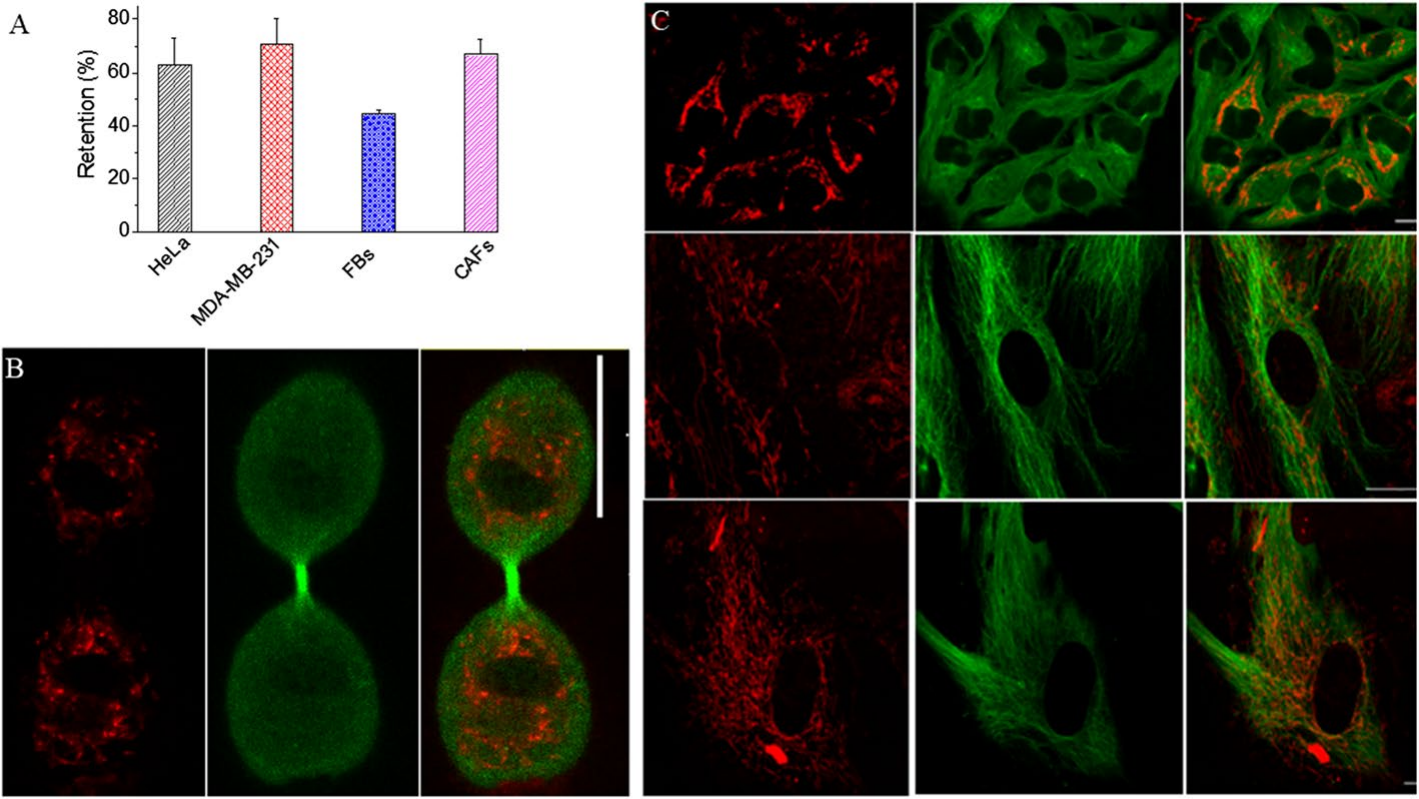
Quantitative and qualitative analysis of cellular retention of GNP_PEG-RGD_. **a** Quantitative data representing percent of retention of NPs. The cells were first incubated with NPs over a time period of 24 h followed by another incubation for 24 h in fresh media to determine the extent of NP retention. **b** Redistribution of GNPs in a parent cell among two daughter cells during cell division. **c** Confocal images of HeLa (first row), FBs (second row), and CAFs (third row) displaying distribution of GNPs (first column; marked in red), MT network (second column, marked in green), and merged image (third column) corresponding to GNPs and MTs. Experiments were repeated three times and the data presented are the average. The error bars represent standard error. Scale bar = 20 μm

The processing of internalized GNPs involves many steps (Huotari and Helenius 2011). For example, NPs first encounter membrane-bound intracellular vesicles called early endosomes once they are internalized by the cells through the endocytosis process. These endosomal vesicles are categorized into three types: early endosomes, late endosomes and recycling endosomes. Early endosomes ferry the cargo to the desired cellular destination. Part of the cargo such as cell surface receptors is recycled back to the plasma membrane via recycling endosomes. Early endosomes then transform into late endosomes followed by integration with lysosomes to form endolysosomal vesicles. The hydrolytic enzymes contained within these vesicles degrade the trapped NPs. We looked at the distribution of NPs in endolysosomal vesicles within the MT network of the cell. According to Fig. 5, there were fewer lysosomes in control cells compared to cells treated with GNPs. This increase in the number of endolysosomal vesicles in cells treated with NPs could be due to the additional processing necessary. After an incubation period of 24 h, most of the NPs were in endolysosomal vesicles; however, there were some NPs still in endosomal vesicles. This is due to the fact that NPs are constantly taken up, processed, and removed by cells, resulting in this distribution. Our results are consistent with previous studies where most of the NPs were in endolysosomal vesicles after 24 h of incubation (Chithrani et al. 2009; Foroozandeh and Aziz 2018). According to Fig. 5b, there was a significant increase in number of endolysosomal vesicles in tumor cells and CAFs compared to FBs. This could be due to increase in NP uptake (see Fig. 3) in cancer cells and CAFs compared to FBs. Considering the fact that FBs turned into CAFs to support tumor growth, we also looked at whether there is a change in the number of mitochondria present in FBs vs CAFs (Additional file 1: S7). Based on the imaging data, there was no significant difference in the presence of mitochondria in FBs vs CAFs. The ability of cells to retain NPs can play a significant role in nanotechnology-based applications in cancer therapy (Srinivasan et al. 2015). We looked at the potential of retaining GNPs within these three cell types once the media containing GNPs was replaced with fresh media for a duration of 24 h. Our quantification results in Fig. 6a demonstrate that the proportion of GNPs retained in HeLa and CAFs was higher compared to FBs. The observed drop in GNP content could be due to exocytosis or redistribution of NPs via division (Chithrani and Chan 2007; Kim et al. 2011a). For example, redistribution of GNPs in a parent cell between two daughter cells is given in Fig. 6b.

This would result in lower number of GNPs in each daughter cell compared to the original parent cell. Both cancer cells and CAFs were able to retain over 60% of internalized NPs even after 24 h. In the case of FBs, the retention of NPs was approximately 40%, which is much lower compared to both CAFs and cancer cells. Qualitative data presented in Fig. 6c support the quantification data given in Fig. 6a. A significant number of NPs are still present in cancer cells and CAFs compared to normal FBs (see Additional file 1: S8–S11 for data corresponding to additional data all 4 cell lines studied). Thus, according to our uptake and retention studies, cancer cells and CAFs have both a significantly higher uptake and longer retention compared to FBs. This could be ideal for the translation of GNPs as drug carriers and radiation sensitizers into current cancer therapy, since the observed behavior of the cancer-associated cells compared to the normal FBs is conducive to reducing normal tissue toxicity.

### Cell proliferation and DNA damage in the presence of NPs

**Fig. 7:**
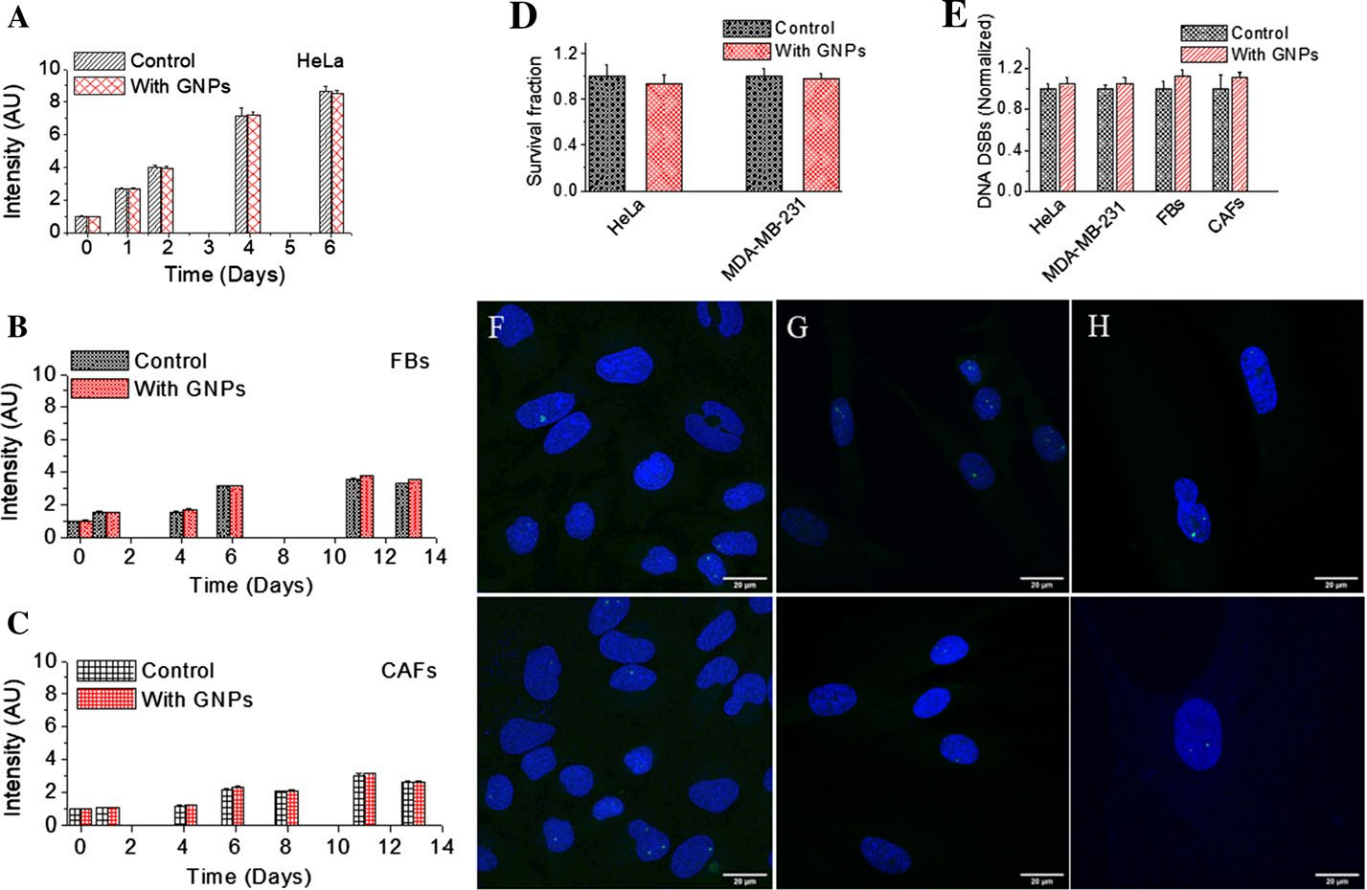
Evaluation of toxicity introduced by GNPs via probing of proliferation and DNA damage. **a–c** Cell proliferation as a function of time for HeLa, FBs, and CAFs, respectively. **d** Cell survival fraction measured using a clonogenic assay for HeLa and MDA-MB-231. **e** Comparison of DNA double strand breaks (DSBs) between control cells and ones treated with GNPs as measured using 53BP1 foci. **f–h** Projected confocal images of HeLa (first column), FBs (second column), and CAFs (third column), respectively. Nuclei and 53BP1 foci are marked in blue and green, respectively. Experiments were repeated three times and the data presented are the average of at least 50 nuclei. The error bars represent standard deviation. Scale bar = 20 µm

The ultimate goal of using NP as a drug delivery system or radiosensitizer is to increase the therapeutic ratio, or the margin between the dose needed for clinical efficacy and the dose inducing adverse side effects such as toxicity (De Jong and Borm 2008). To yield this full potential of NPs in cancer therapy, it is necessary to evaluate the damage introduced to normal cells *vs* cancer cells. We assessed the toxicity introduced by NPs through monitoring cell proliferation and assessing DNA damage. It is important to mention again that the GNP complex used for the study is fully compatible for future in vivo studies followed by clinical studies, and the concentration utilized is also clinically feasible (Schuemann et al. 2016; Yang et al. 2018a; Zhang et al. 2012). Hence, our results provide meaningful data for designing future experiments. Proliferation of cells was monitored to measure any effect GNPs would have on the growth pattern and the results are given in Fig. 7a–c for HeLa, FBs, and CAFs, respectively. It was important to notice that there was no significant toxicity induced by the GNPs to FBs or cancer-associated cells (HeLa and CAFs). We also fitted the experimental data shown in Fig. 7a–c to calculate the doubling time ($T_{d}$) for each cell line (Additional file 1: S12). Based on the fitted curves, $T_{d}$ for HeLa, FBs, and CAFs were 19.5, 49.7 and 77 h, respectively (p = 0.009) and the values are in agreement with previous literature (Liberato et al. 2018; Puck et al. 1956; Zhang et al. 2012). According to our fitted data, there was no significant difference in the growth with the addition of GNPs relative to control in all three cell lines. We also looked at long-term effects of NPs on cell growth using a clonogenic assay. There was no introduced toxicity due to GNPs for both HeLa and MDA-MB-231 (Fig. 7d). It was very difficult to carry out clonogenic assay for FBs and CAFs since their $T_{d}$ was much longer and they did not form consistent colonies. Furthermore, there was also no significant increase in DNA damage with the addition of GNPs in any cell line (see Fig. 7e, f–h). We probed the most lethal damage to DNA, which is double stand breaks (DSBs), using an antibody targeted towards one of the repair proteins, 53BP1. The number of 53BP1 foci in cells treated with GNPs was compared to the control (see Fig. 7e, f–h). Thus, it can be concluded the GNP complexes used in this study themselves, i.e., without radiation, do not have a toxic effect on either of the cell lines.

## Conclusion

This study unveils the differential cross section of NP uptake, processing, retention, and toxicity across key cell components of the TME (tumor cells, FBs, and CAFs) for the first time (see Fig. 1). In this study, we used GNPs of 15 nm diameter which were functionalized with both PEG molecules and a peptide containing integrin-binding domain, RGD. Both CAFs and FBs play a significant role in tumor growth: FBs can exert diverse suppressive functions against cancer initiating and metastatic cells in order to suppress tumor progression while CAFs could promote tumor growth. In order to build an ideal NP-based therapeutic platform to battle cancer, we need to eradicate both cancer cells and CAFs while keeping the damage to a minimum in FBs. Results of our study showed that cellular uptake of GNPs per unit cell volume for HeLa (tumor cells) and CAFs was over 25- and 10-fold higher compared to the FBs. However, the significantly higher presence of GNPs within cells did not introduce any additional toxicity, based on our proliferation and DNA damage results. Further, FBs have the least ability to retain the NPs within the cell body as compared to tumor cells and CAFs. The higher NP uptake and retention in tumor cells and CAFs as compared to FBs is very encouraging and significant for their potential use in future clinical trials. A recent study clearly showed the bridge between the MT network and NP transport, using one of the most common anticancer drugs, docetaxel, which was used to manipulate MTs for trapping NPs closer to the nucleus for a longer period of time (Bannister et al. 2019). This resulted in higher radiation dose enhancement during RT and finally producing synergistic therapeutic outcome in GNP-mediated chemoradiation. Due to the higher number of GNPs present in tumor cells and CAFs compared to normal FBs, we propose to exploit the MT network using such chemotherapeutic drugs in designing smart NP-based medicine for optimized outcome in therapeutics. Furthermore, over 20 nanotechnology-based therapeutic products have been approved for clinical use in the past two decades (Miao and Huang 2015). Considering clinical trials that have been concluded successfully using GNPs either as a drug delivery vehicle or as a photothermal agent, GNP-mediated cancer therapeutics with minimum side effects are on the horizon for cancer patients (Libutti et al. 2010; Rastinehad et al. 2019; Schuemann et al. 2016). One of the limitations in this study is the use of one cell line each from normal FB and CAF cell line. Our future studies will extend to many patient-derived FBs and CAFs in order to make predictions in a more diverse and relevant population.

## Materials and methods

### Preparation of GNPs

Citrate reduction method was used to prepare GNPs of diameter ~ 15 nm (Kimling et al. 2006). In summary, 300 µL of 1% chloroauric acid (${\text{HAuCl}}_{4}$) was added to 30 mL of double distilled water followed by heating while stirring. 1 mL of 1% sodium citrate tribasic dihydrate $\left( {{\text{HOC}}\left( {\text{COONa}} \right)\left( {{\text{CH}}_{2} {\text{COONa}}} \right)_{2} \cdot2{\text{H}}_{2} {\text{O}}} \right)$ was added to the mixture once it reached the boiling point and kept stirring until the color of the mixture turned a ruby red. The solution was brought back to room temperature while stirring.

### Surface functionalization of GNPs

As illustrated in Fig. 2, GNPs were surface functionalized with both PEG (2 kDa PEG-thiol) and a peptide containing integrin-binding domain, RGD (RGD peptide: N ${\text{H}}_{2}$-Cys-Lys-Lys–Lys-Lys–Lys-Lys-Gly-Gly-**Arg-Gly-Asp**-Met-Phe-Gly-COOH). The GNPs were first surface functionalized with PEG at a ratio of 1 PEG molecule per ${\text{nm}}^{2}$ of surface area, assuming a perfect sphere (${\text{GNP}}_{\text{PEG}}$). For optical imaging,${\text{GNP}}_{{{\text{PEG}} - {\text{Cy}}5}}$ was synthesized with a mix of the 2-kDa PEG and a 3.2-kDa PEG-thiol-Cy5 complex in equal proportions. To prepare ${\text{GNP}}_{{{\text{PEG}} - {\text{RGD}}}}$, RGD peptide was added to mixture containing ${\text{GNP}}_{\text{PEG}}$ at a ratio of 1 peptide molecule per every 2 PEG molecules (${\text{GNP}}_{{{\text{PEG}} - {\text{RGD}}}}$).

GNP complexes were characterized using via transmission electron microscopy (TEM), ultraviolet–visible (UV–Vis) spectrometry (Perkin Elmer $\lambda$ 365 Spectrophotometer), dynamic light scattering (DLS; Anton Paar LiteSizer 500), and $\zeta$-potential (Anton Paar LiteSizer 500). We also used darkfield microscopy and hyper spectral imaging (HSI; CytoViva) for characterization of GNPs. For TEM imaging, GNPs were placed on a grid and dried before imaging. We used cuvettes for UV, DLS, and zeta potential measurements of GNP complexes in aqueous medium. GNP complexes were placed on cover slips and dried before mounting them on microscope glass slides for darkfield and HSI imaging.

### Cellular uptake and retention of gold nanoparticle complexes

HeLa, MDA-MB-231, normal fibroblast, and cancer-associated fibroblasts were obtained from ATCC in 2019 and the catalog numbers are CCL-2, HTB-26, CRL-7636, and CRL 7637, respectively. Cells were cultured in high glucose Dulbecco’s modified Eagle medium (DMEM; Gibco) supplemented with 10% fetal bovine serum (FBS; Gibco), 1% penicillin/streptomycin (Gibco), and 4 mM of GlutaMax (Gibco). For optical imaging experiments, colorless media (FluoroBrite DMEM (Gibco)) was supplemented with 10% FBS and 1% penicillin/streptomycin. We used CellLight Tubulin-GFP (BacMam 2.0, Thermo-Fisher) for staining microtubules. For live-cell imaging, cells were grown on 3 cm coverslip-bottom dishes (MatTek). For dark field imaging, cells were grown on cover slips and fixed after the treatment using paraformaldehyde (PFA; Sigma Aldrich). Trypsin–EDTA(Gibco) was used for cell removal from dishes for quantification studies. For confocal experiments, FluoroBrite DMEM (Gibco) was supplemented with 10% FBS and 1% penicillin/streptomycin after staining with CellLight Tubulin-GFP (BacMam 2.0, Thermo-Fisher), while the cells were grown on 3 cm coverslip-bottom dishes supplied by MatTek. Cells were fixed using paraformaldehyde (PFA; Sigma Aldrich).

For determining the dynamics of GNP uptake, 1 × 10^4^ cells were plated in 6-well plates and left for 24 h to ensure adherence in the incubator. After cells were adhered to the bottom of the dishes, they were all incubated with ${\text{GNP}}_{{{\text{PEG}} - {\text{RGD}}}}$ at 0.2 nM concentration in media for 1, 4, 8, and 24 h at 37 ͦC with 5% $O_{2}$. After specific NP incubation time period, cells were washed with phosphate buffered saline (PBS) three times, trypsinized, and counted using a Coulter Counter (Z2 Coulter; Beckman Coulter) for the quantification purposes.

For the retention study, cells were first incubated with ${\text{GNP}}_{{{\text{PEG}} - {\text{RGD}}}}$ for 24 h time period. After the incubation with GNPs, cells were washed with PBS three times, added fresh media, and left in the incubator for a 24-h time period. Following the incubation with fresh media, cells were washed with PBS, removed from the dishes, and counted for quantification studies.

To measure the gold content for each condition, the cells were treated with 65% aqua regia (3:1 ratio of ${\text{HCl}}:{\text{HNO}}_{3} \left( {\text{VWR}} \right)) {\text{ in a }}200 ^{\text{o}} {\text{C}}$ mineral oil bath for a minimum 1 h. Small amounts of hydrogen peroxide were added afterwards to ensure complete digestion of the cells and GNPs. These samples were then diluted down to 2.5% *v/v* acid content in deionized water and the gold content was quantified using inductively coupled plasma mass spectrometry (ICP-MS; Agilent 8800 Triple Quadrupole).

### Preparation of cells for imaging

We used both darkfield and confocal imaging to determine the distribution of GNPs. For darkfield imaging, all cell lines were plated on coverslips placed on the bottom of 6 well dishes. The cells were treated with ${\text{GNP}}_{{{\text{PEG}} - {\text{RGD}}}}$ for 24 h to determine the extent of endocytosis. Upon completion of NP incubation, the cells were rinsed three times with PBS and fixed using 4% paraformaldehyde (PFA) for 20 min at 37 ͦC. The cover slips were washed with PBS, removed from each well, and mounted to a glass slide using Prolong Glass Antifade Mountant. Each sample was imaged using darkfield microscopy and HSI (CytoViva) under a 60X objective.

Live-cell imaging was performed using confocal microscopy (Zeiss LSM 980) using a 60X oil immersion lens. For confocal imaging, ${\text{GNP}}_{{{\text{PEG}} - {\text{RGD}}}}$ complexes had PEG-Cy5 (excitation 633 nm, emission filter 650 nm LP) conjugated as previously mentioned. To see general structure of the cell, microtubules (MTs) were stained with a viral transfection stain (CellLight Tubulin-GFP), which contains DNA coding for an α-tubulin/GFP construct. For live-cell confocal imaging, cells were plated on 3 cm coverslip-bottomed dishes in FluoroBrite media. For staining MTs, the cells were incubated in the viral stain for  > 24 h prior to treatment with fluorescent ${\text{GNP}}_{{{\text{PEG}} - {\text{RGD}}}} .$ After NP incubation, the cells were imaged after 24 h of endocytosis. To determine the retention, cells were first incubated with GNPs for 24 h, removed the media, added fresh media, and incubated for 24 h. All imaging parameters (acquisition settings) used between experiments was maintained constant.

### Immunofluorescence assay

Once the cells were adhered to glass coverslips in 6-well plates, fresh media with or without (control) GNPs were added followed by a 24-h incubation time period. After the incubation time period, the cells were washed with PBS three times and fixed with 4% PFA for 5 min at room temperature followed by two PBS washes for 5 min each. The cells were then treated with 2% BSA/0.1% Triton-X in PBS for 20 min. The two primary antibodies γH2AX and 53BP1 were diluted 1:200 in 0.5% BSA/0.1% Triton-X/PBS, while the two secondary antibodies were diluted 1:500 in 0.5% BSA/0.1% Triton-X/PBS. The coverslips were first incubated with a combination of the two primary antibodies on parafilm for 1 h, followed by washing with PBS three times. The cells were then rinsed twice with 0.5% BSA/0.175% Tween-20/PBS for 5-min time durations. Finally, the cover slips were incubated with secondary antibodies in the dark for 30 min. After the incubation time period, the cells were rinsed in PBS, dried, and mounted to glass coverslips with Prolong Glass.

### Statistical analysis

A *t* test correcting for multiple comparisons using the Holm–Sidak method was performed using GraphPad Prism 8. A p-value < 0.05 was considered statistically significant. All experiments were repeated three times and the data presented is the average.

## Supplementary information

**Additional file 1**: Supplementary Figures.

## Data Availability

Supplementary material is available.

## Abbreviations

NPs: Nanoparticles
GNPs: Gold nanoparticles
RT: Radiotherapy
CT: Chemotherapy
TME: Tumor microenvironment
FBs: Fibroblasts
CAFs: Cancer-associated fibroblasts
ICP-MS: Inductively coupled plasma mass spectroscopy
TEM: Transmission electron microscopy
HSI: Hyperspectral imaging
UV: Ultraviolet
DSBs: Double strand breaks
DMEM: Dulbecco’s modified Eagle’s medium
PBS: Phosphate buffered saline
