# Supplementary material for: Elucidating the fate of nanoparticles among key cell components of the tumor microenvironment for promoting cancer nanotechnology
Source: Cancer Nanotechnol. 2020 Aug 18;11(1):8. doi: 10.1186/s12645-020-00064-6 (PMC7437649; doi:10.1186/s12645-020-00064-6)

Supplementary section S1: Darkfield images of triple negative breast cancer cell line, MDA-MB-231

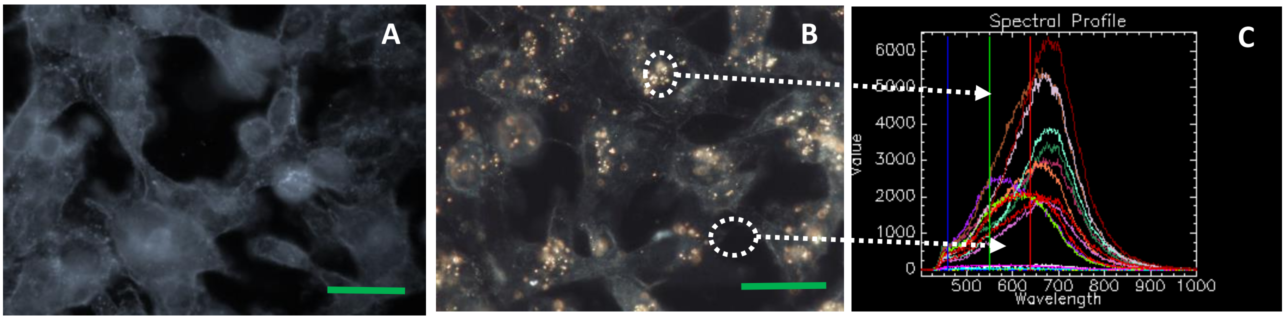

Supplement Figure S1. Darkfield images of MDA-MB-231 control cells (A) and ones with internalized GNPs (B). (C) Spectra collected from GNP clusters and background in MDA-MB-231 cells (B). The error bars represent standard error. Scale bar = 20 µm.

Supplementary section S2: Cellular uptake of as made GNPs, GNP_{PEG}, and $GNP_{PEG\text{-}RGD}$ in HeLa, normal FBs, and CAFs

It is known that adding PEG reduces the uptake of as-made GNPs. The first row of Figure S1 shows the images of HeLa, FBs, and CAFs when incubated with as-made GNPs for a period of 24 hrs, respectively. Addition of PEG molecules onto as-made GNPs resulted in a very low uptake and images in second row clearly show the decrease in NP uptake for HeLa, FBs, and CAFs. Addition of RGD peptide onto GNP_{PEG} resulted in a significant increase in uptake of GNPs (see the third row of Fig. S1). Our results are consistent with the previously published work using MDA-MD-231 cell line.

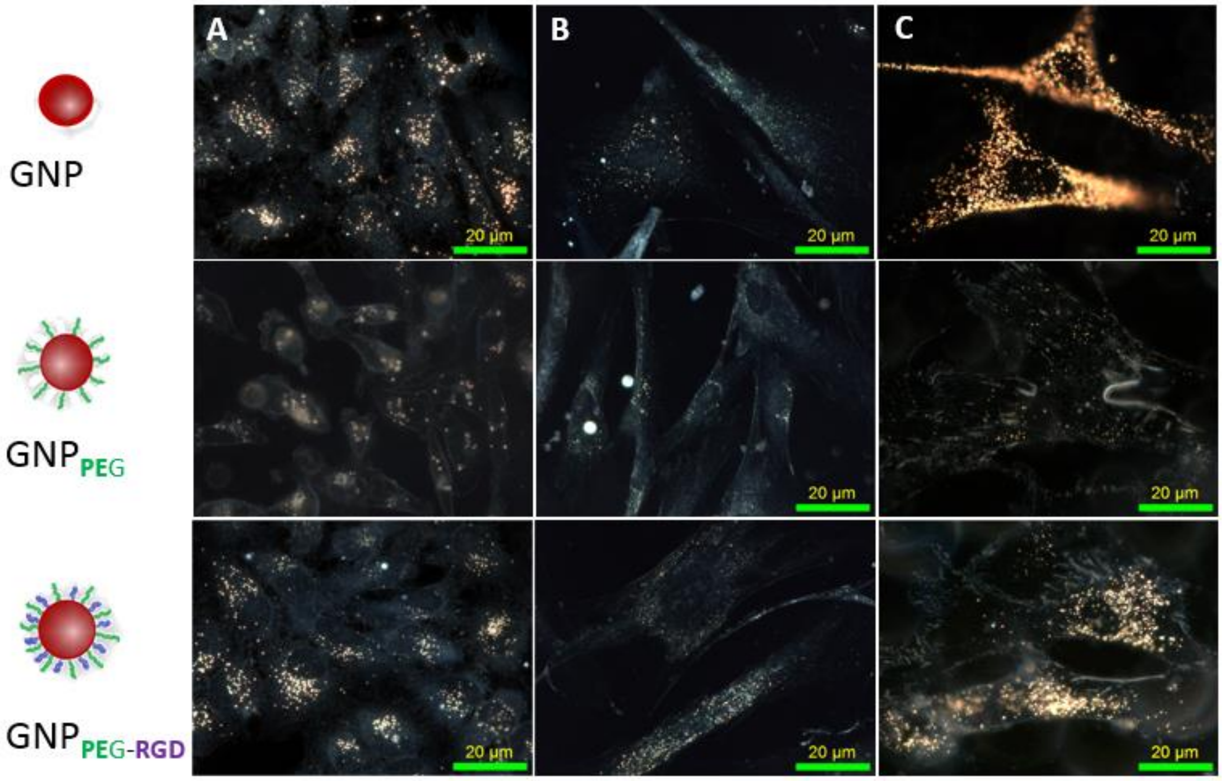

Figure S2. Darkfield images of as-made GNPs (first row), GNP_{PEG} (second row), and $GNP_{PEG\text{-}RGD}$ (third row) in HeLa (column A), normal FBs (column B), and CAFs (column C).

Supplementary section S3: HeLa cells: NP distribution and MT network in a cell population

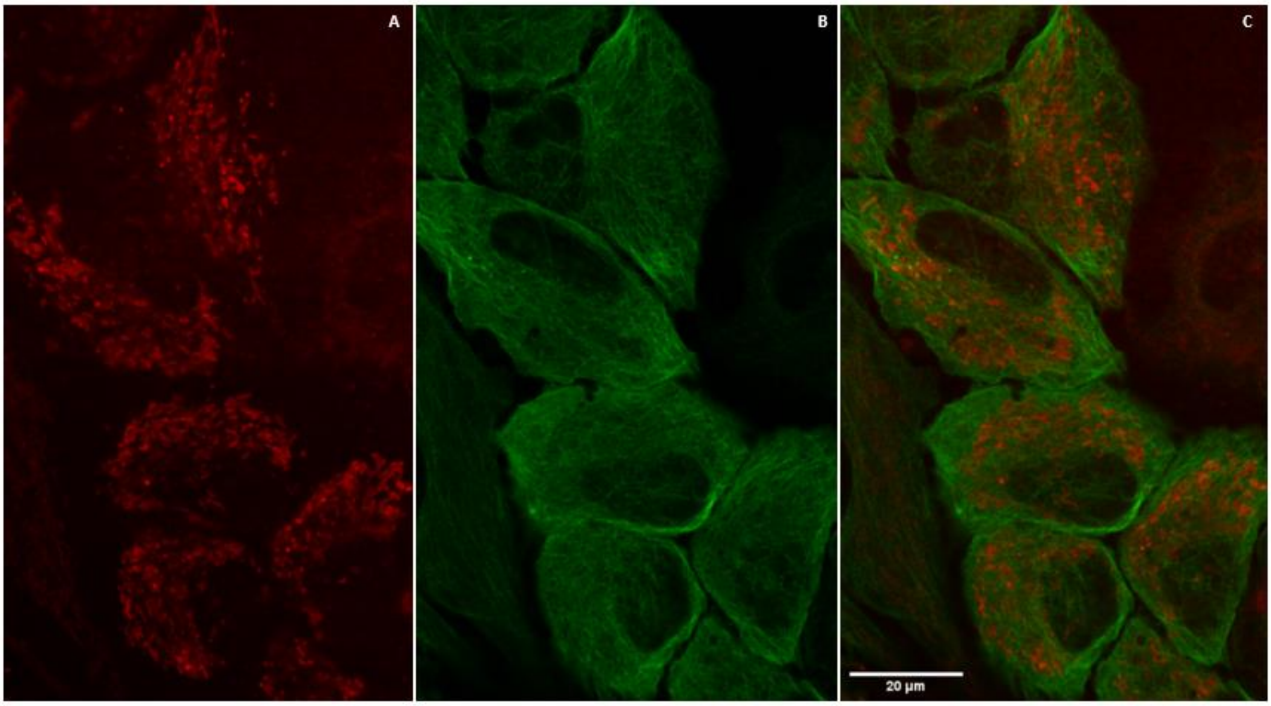

Supplement Figure S3-1. Microtubule (MT) network and distribution of NPs (A-C) Vesicles containing NPs, MT, and merged image, respectively. MTs and GNPs are labelled in green and red, respectively.

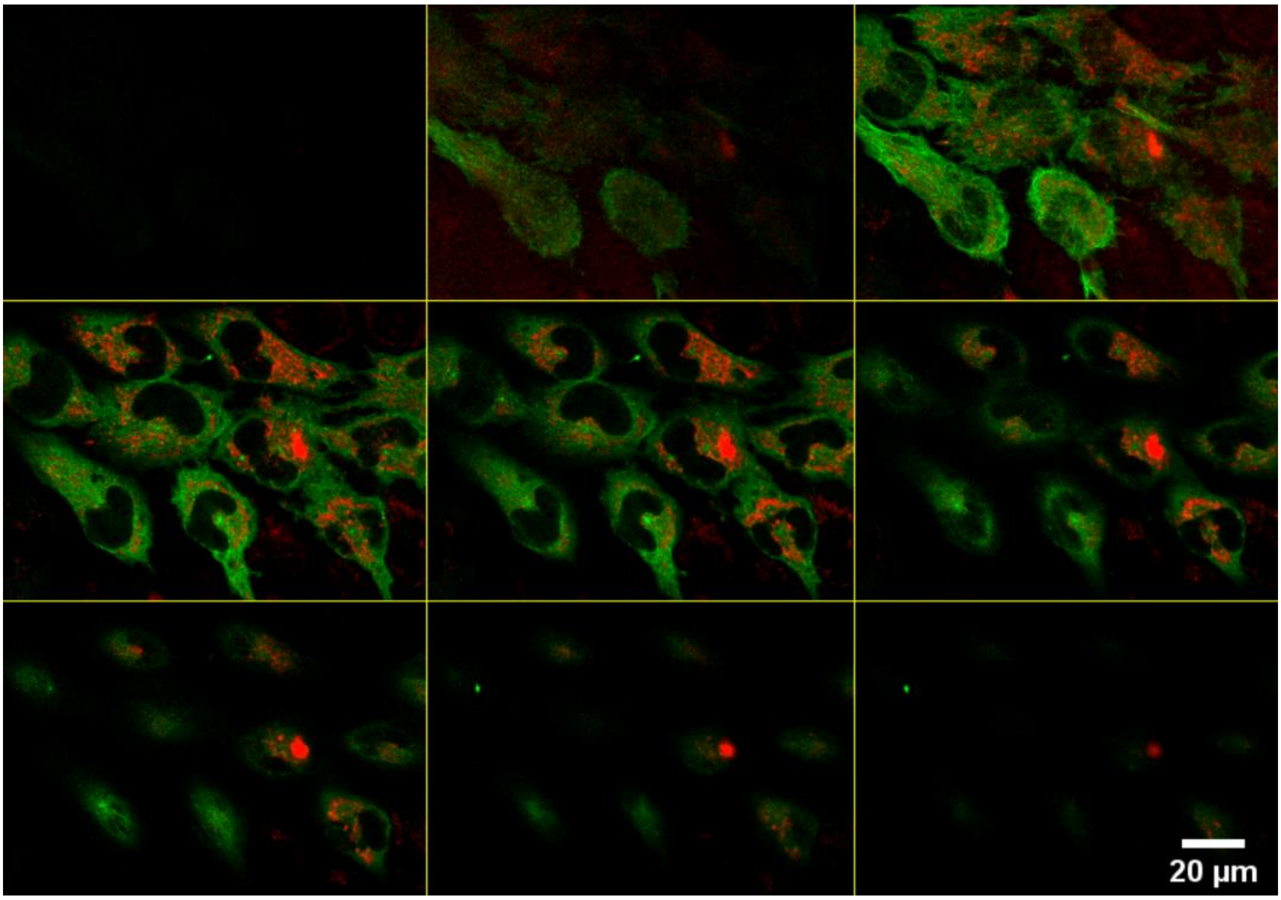

Supplement Figure S3-2. (D) Z-stack showing the distribution of NPs across different planes starting from the adherent plane onwards. The scale bar is 20 µm.

<u>Supplementary section S4:</u> <u>MDA-MB-231: NP distribution and MT network in a cell population</u>

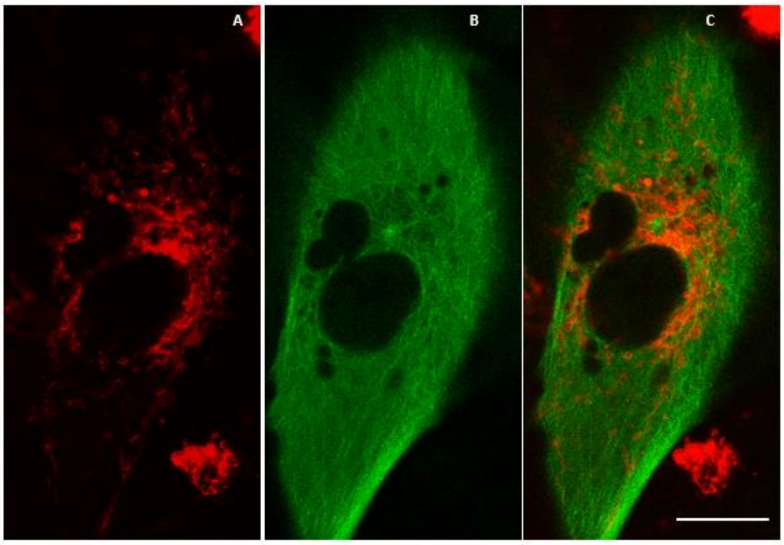

Figure S4-1. Microtubule (MT) network and distribution of NPs within the cell. (A-C) Vesicles containing NPs, MT, and merged image, respectively. MTs and GNPs are labelled in green and red, respectively. The scale bar is 20 µm.

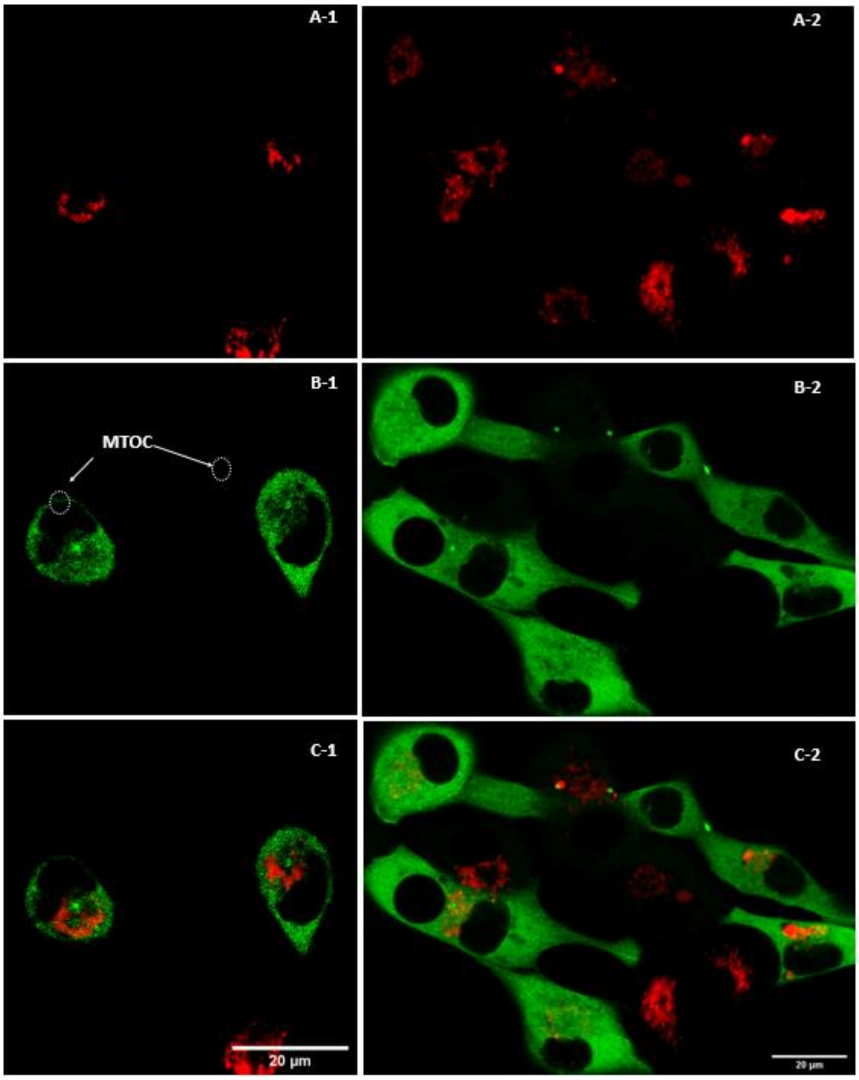

Supplement Figure S4-2. Microtubule (MT) network and distribution of NPs in a cell population. (A-C) Vesicles containing NPs, MT, and merged image, respectively. MTs and GNPs are labelled in green and red, respectively. The scale bar is 20 µm.

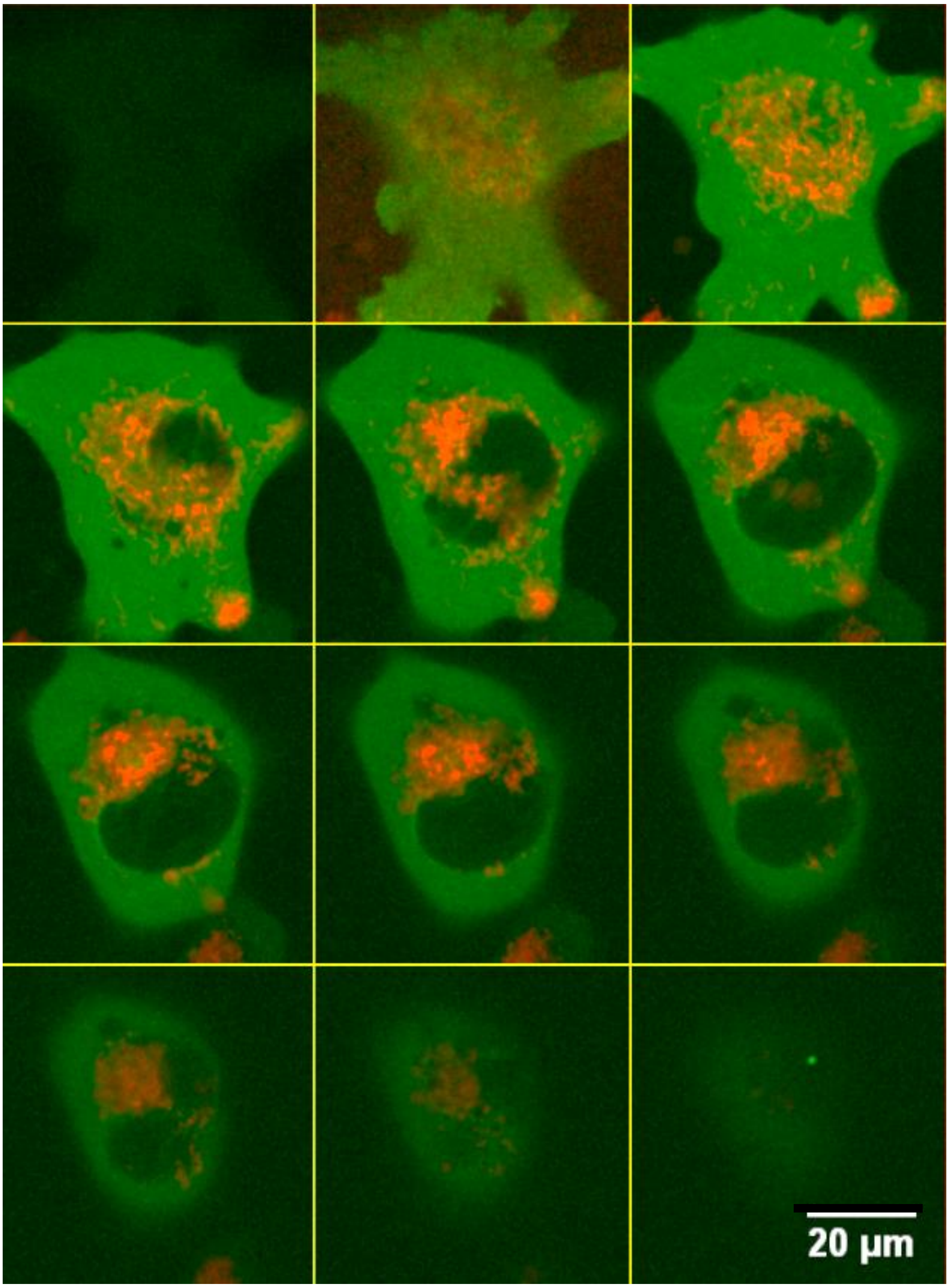

Supplement Figure S4-3. Z-stack showing the distribution of NPs across different planes starting from the adherent plane onwards. The scale bar is 20 µm.

Supplementary section S5: Normal fibroblasts (FBs): NP distribution and MT network in a cell population

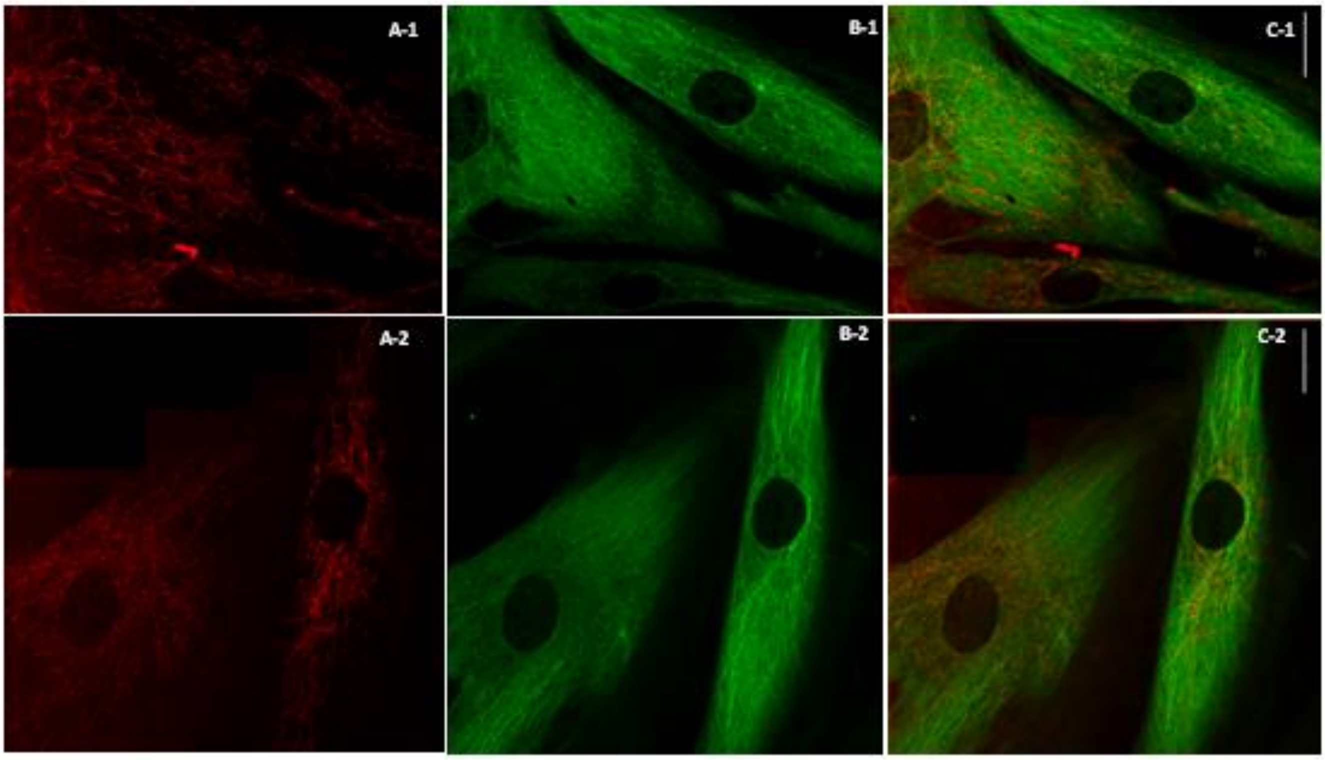

Figure S5-1. Microtubule (MT) network and distribution of NPs within the cell. (A-C) Vesicles containing NPs, MT, and merged image, respectively. MTs and GNPs are labelled in green and red, respectively. The scale bar is 20 µm.

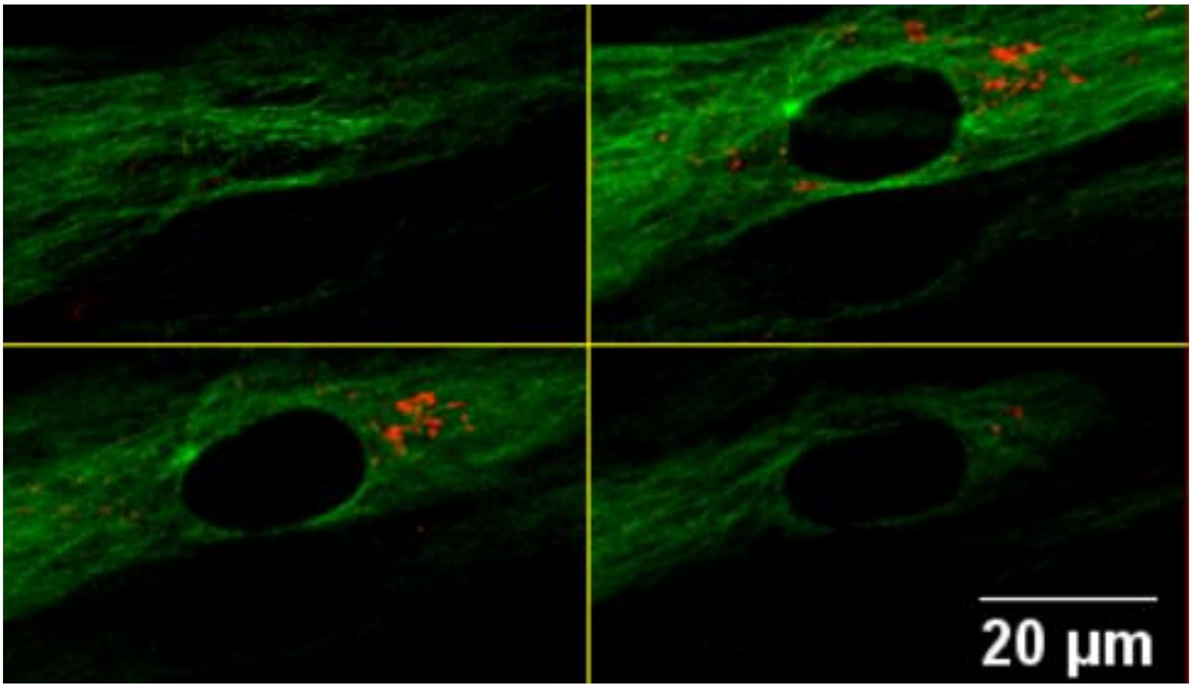

Supplement Figure S5-2. Z-stack showing the distribution of NPs across different planes starting from the adherent plane onwards.

<u>Supplementary section S6:</u> <u>Cancer associate fibroblasts (CAFs): NP distribution and MT network in a cell population</u>

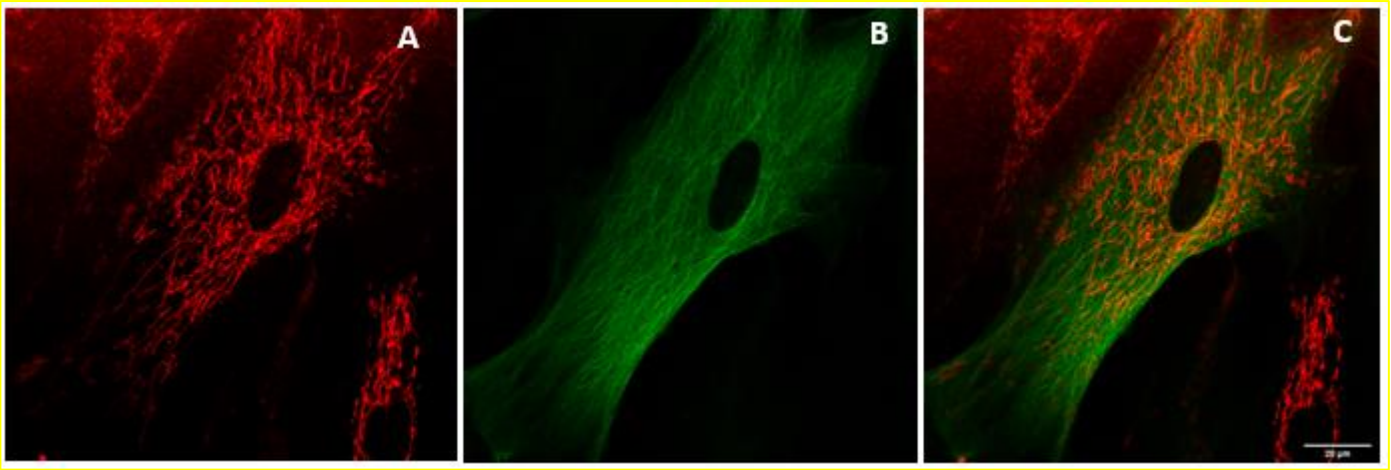

Supplement Figure S6-1. Microtubule (MT) network and distribution of NPs within the cell. (A-C) Vesicles containing NPs, MT, and merged image, respectively. MTs and GNPs are labelled in green and red, respectively. The scale bar is 20 µm.

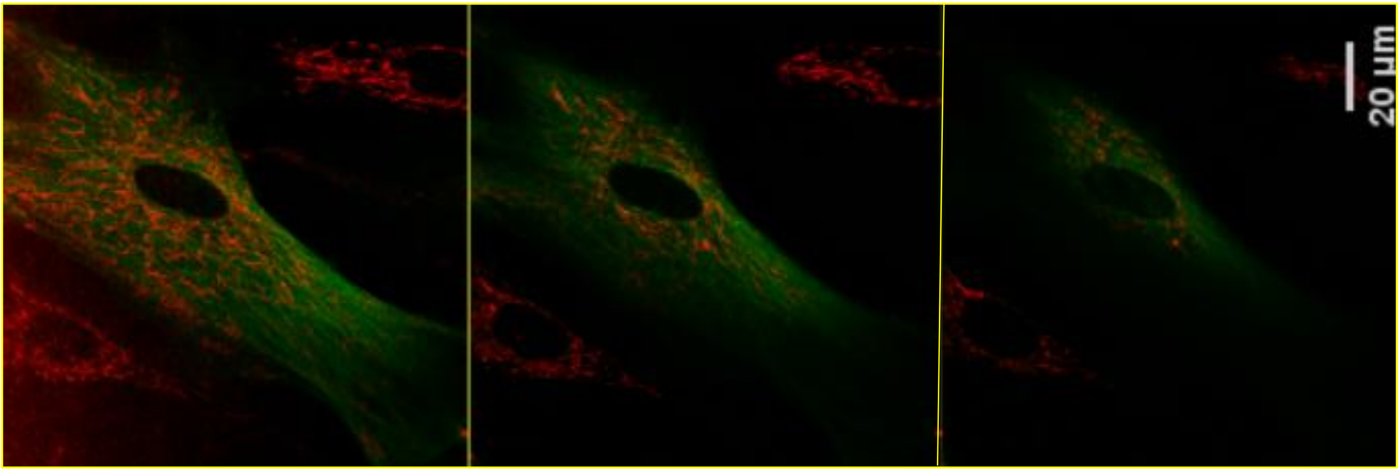

Supplement Figure S6-2. Z-stack showing the distribution of NPs across different planes starting from the adherent plane onwards.

Supplementary section S7: Presence of mitochondria

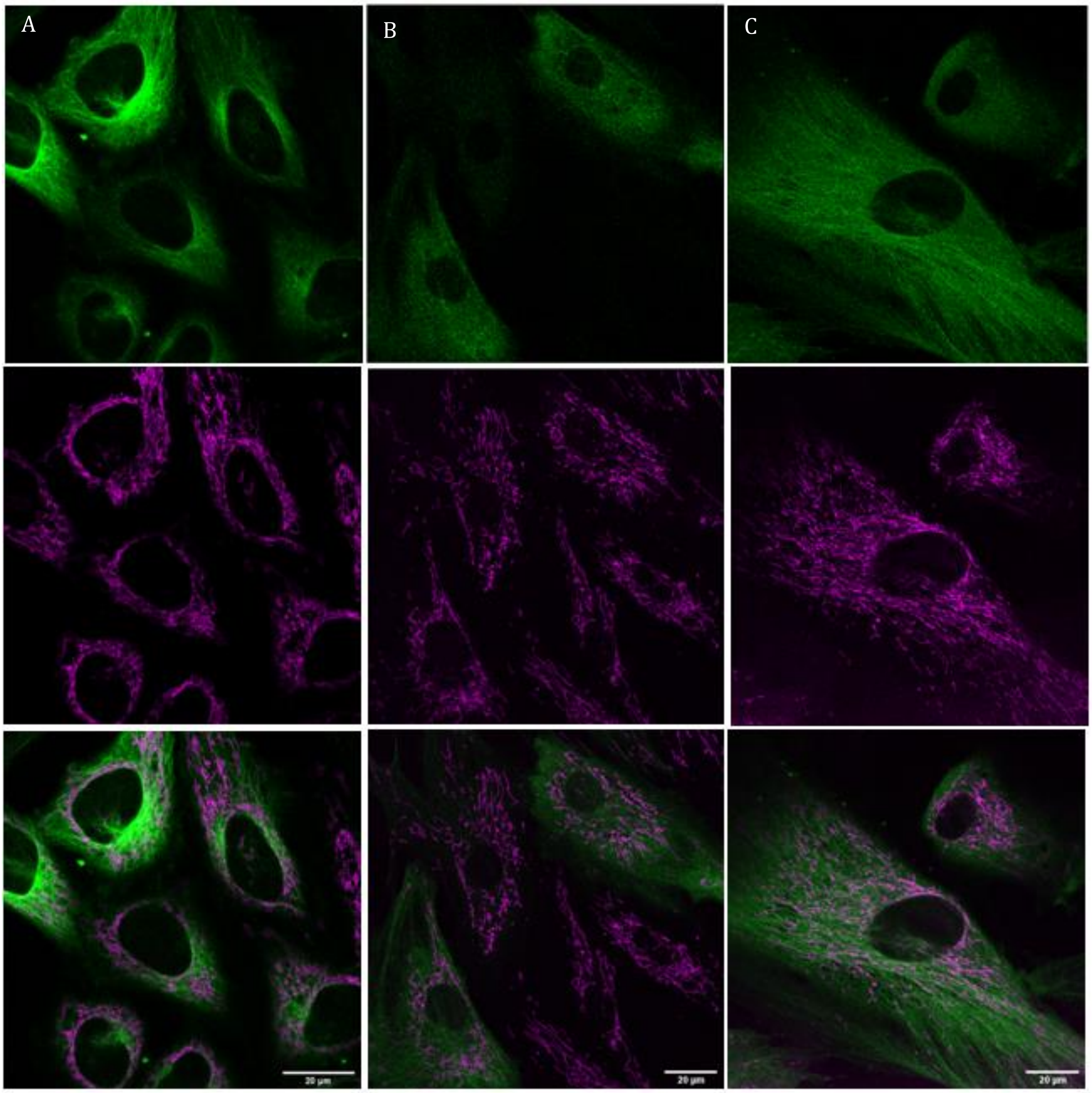

Supplement Figure S7. (A-C) Presence of mitochondria in HeLa, FBs, and CAFs, respectively. First, second, and third rows represent MTs, mictochondira, and the merged images. while right column represent the merge image corresponding to endolysosomal vesicles (yellow) and MTs (green).

<u>Supplementary section S8:</u> <u>Retention of NPs within HeLa cells.</u>

Images show the distribution of GNPs within MDA-MB-231cells along with the Microtubule (MT) network.

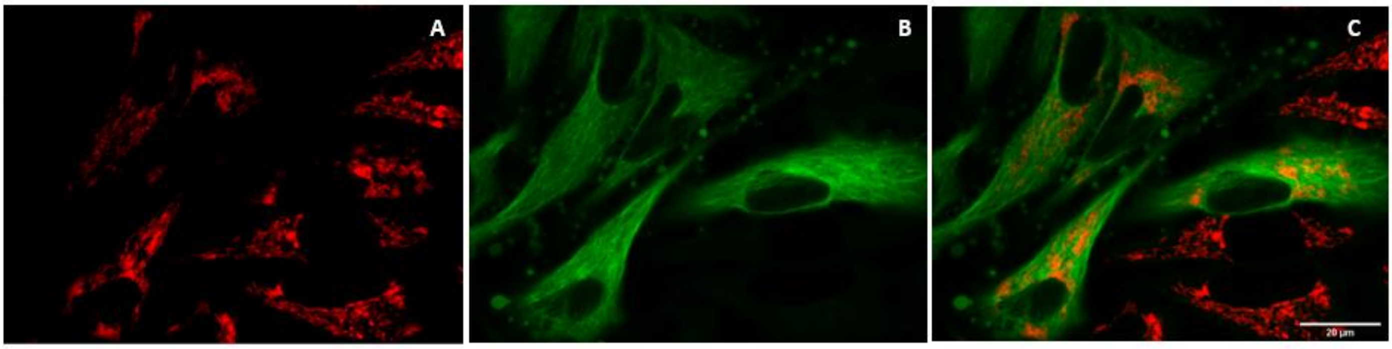

Supplement Figure S8-1. Retention of GNPs in HeLa cells. MTs and GNPs are labelled in green and red, respectively.

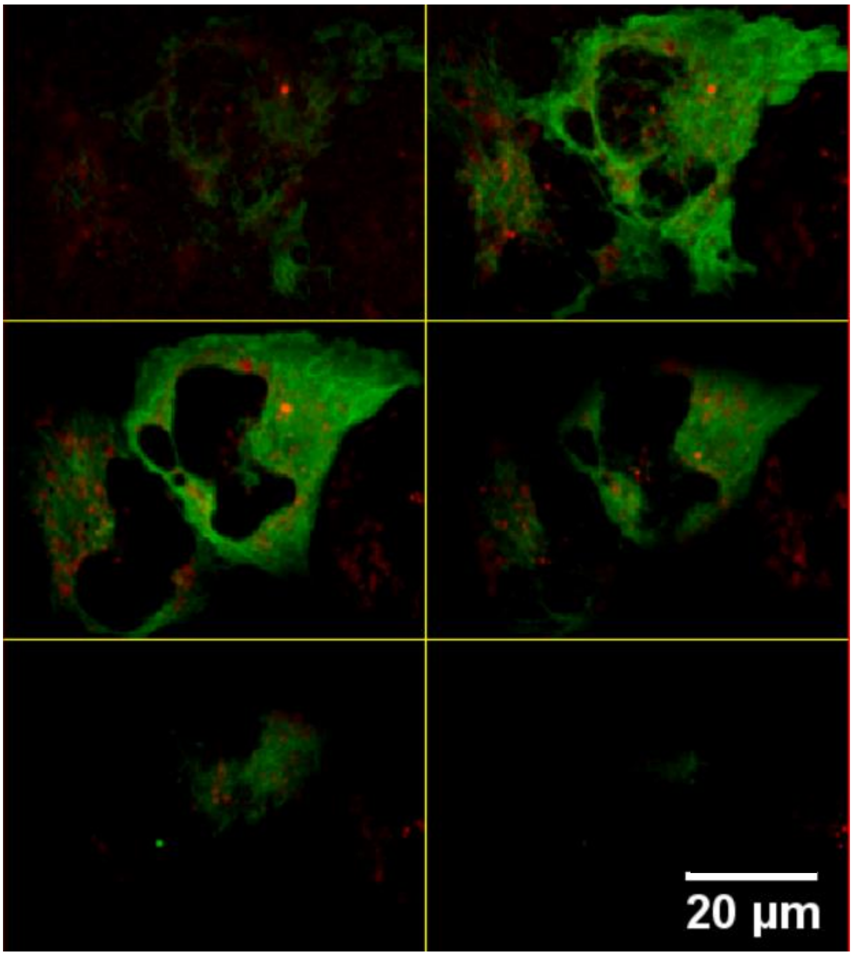

Supplement Figure S8-2. Z-stack showing the distribution of NPs across different planes starting from the adherent plane onwards.

Supplementary section S9: Retention of NPs within MDA-MB-231 cells.

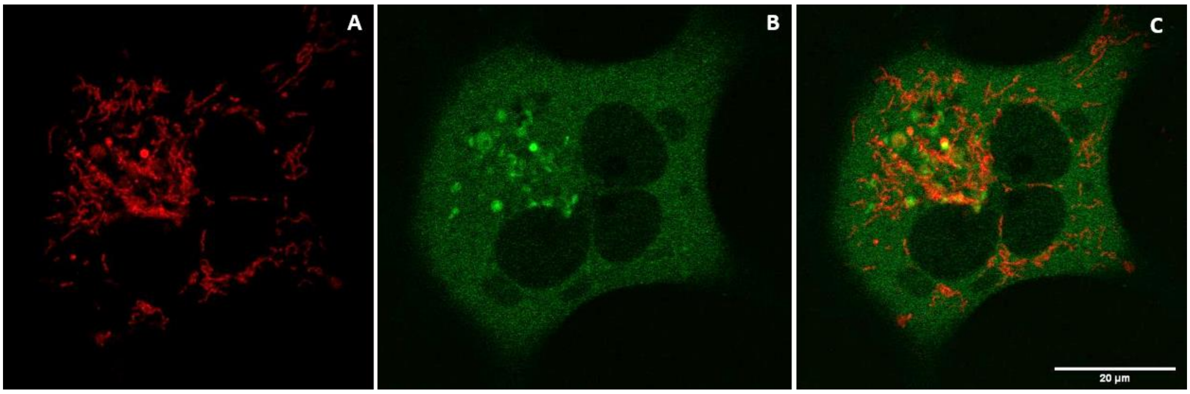

Supplement Figure S9-1. Retention of GNPs within am MDA-MB-231 cell. (A-C) Vesicles containing NPs, MT, and merged image, respectively. MTs and GNPs are labelled in green and red, respectively. The scale bar is 20 µm.

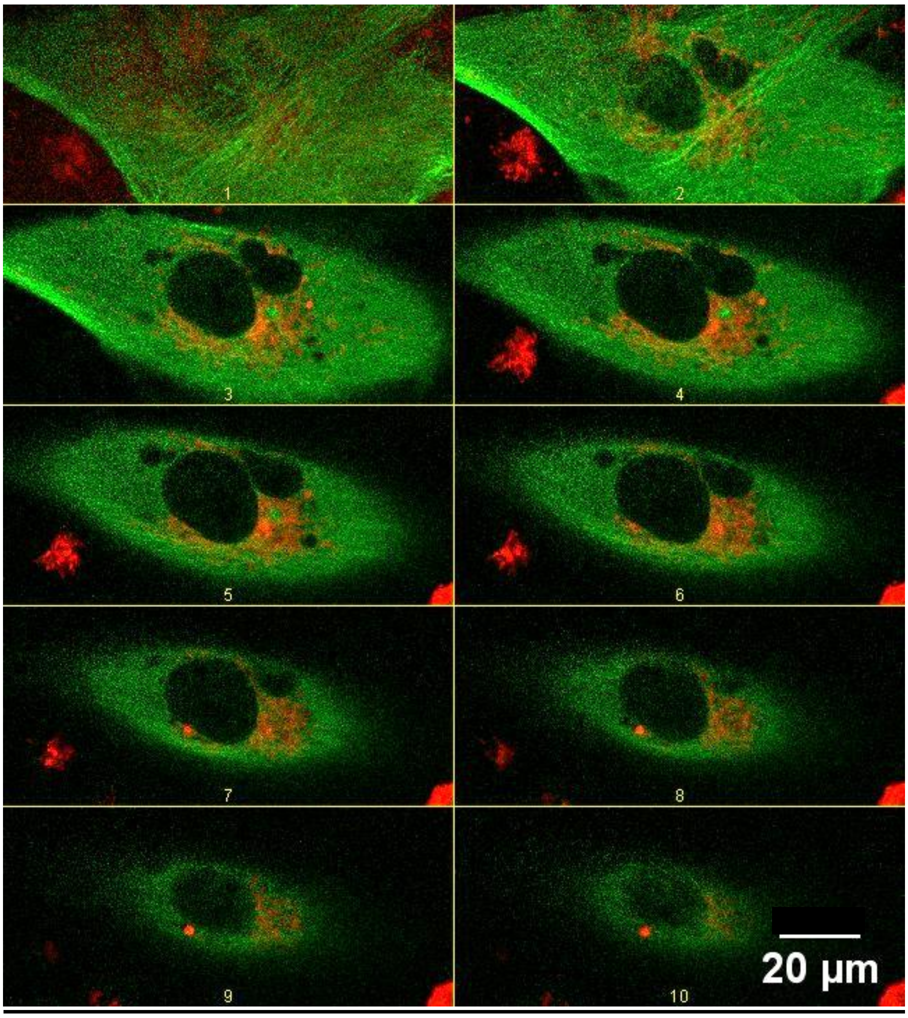

Supplement Figure S9-2. Z-stack showing the distribution of NPs across different planes starting from the adherent plane onwards.

Supplementary section S10: Retention of NPs within normal fibroblast cells.

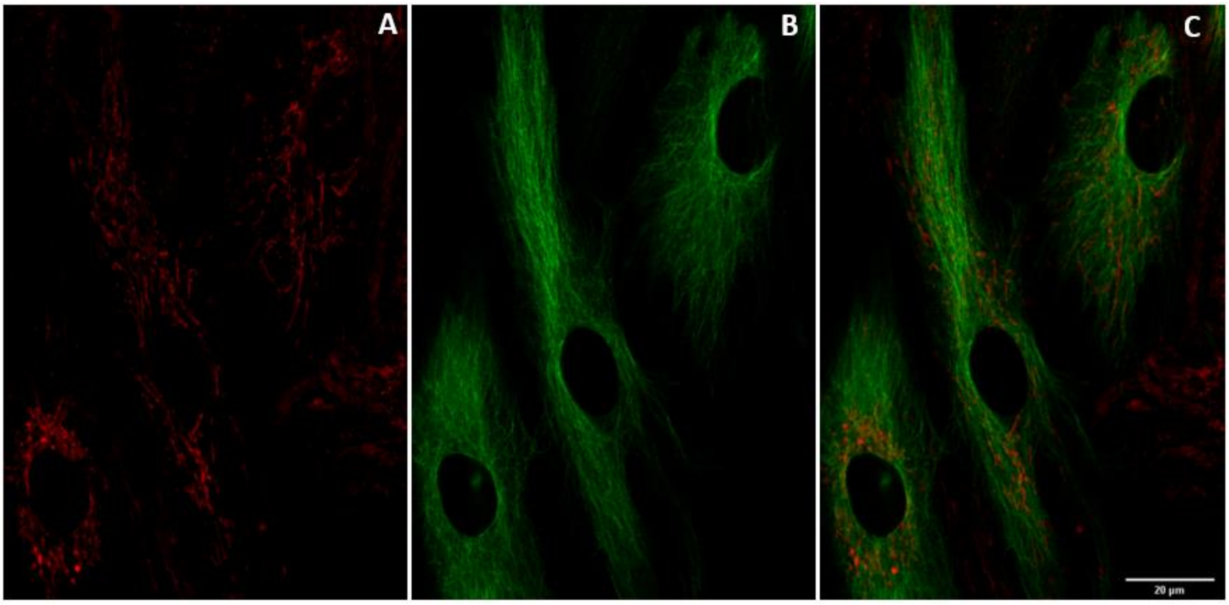

Supplement Figure S10-1. Retention of GNPs. (A-C) Vesicles containing NPs, MT, and merged image, respectively. MTs and GNPs are labelled in green and red, respectively.

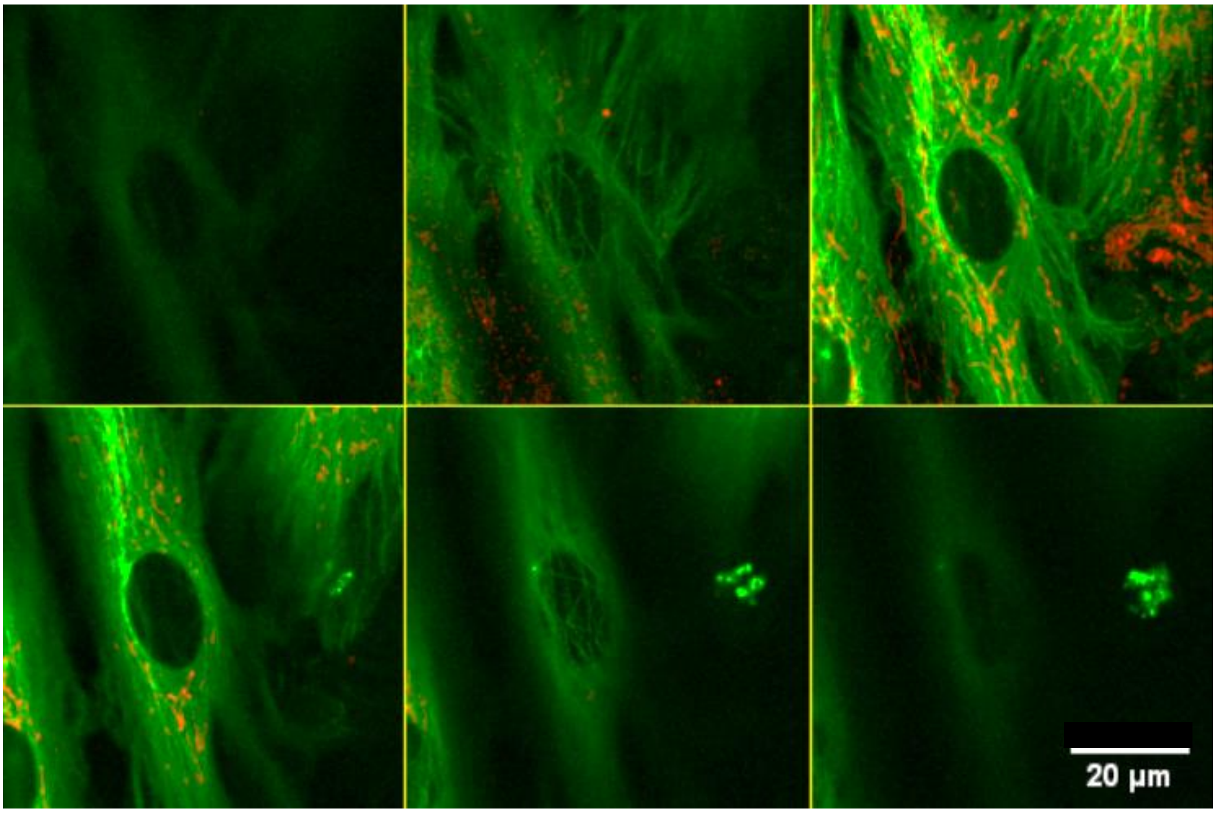

Supplement Figure S10-2. Z-stack showing the distribution of NPs across different planes starting from the adherent plane onwards.

Supplementary section S11:

Retention of NPs within normal cancer associated fibroblast cells.

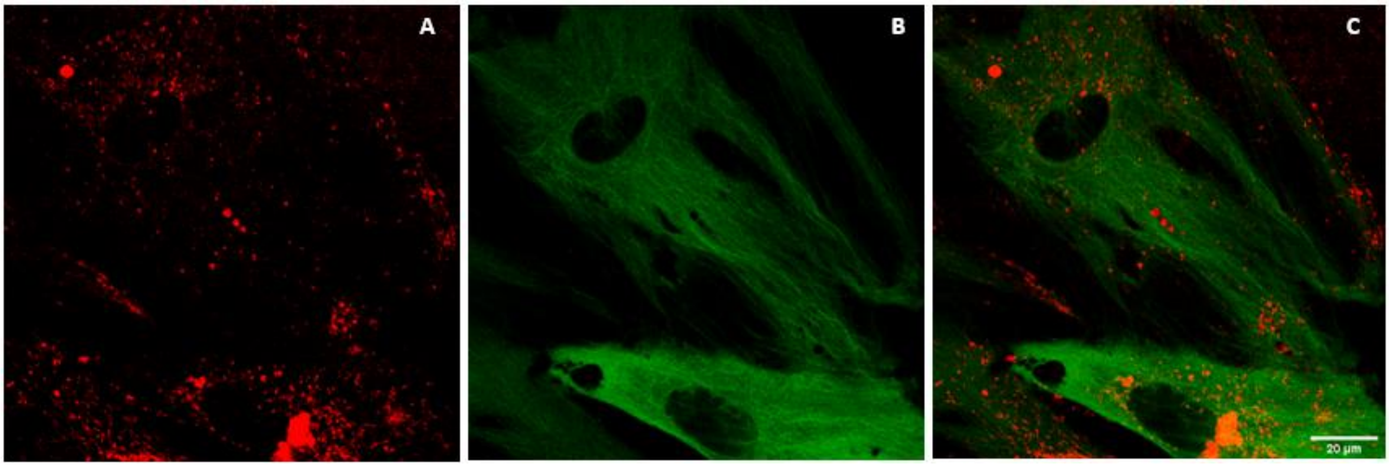

Supplement Figure S11-1. Retention of GNPs in HeLa cells. MTs and GNPs are labelled in green and red, respectively.

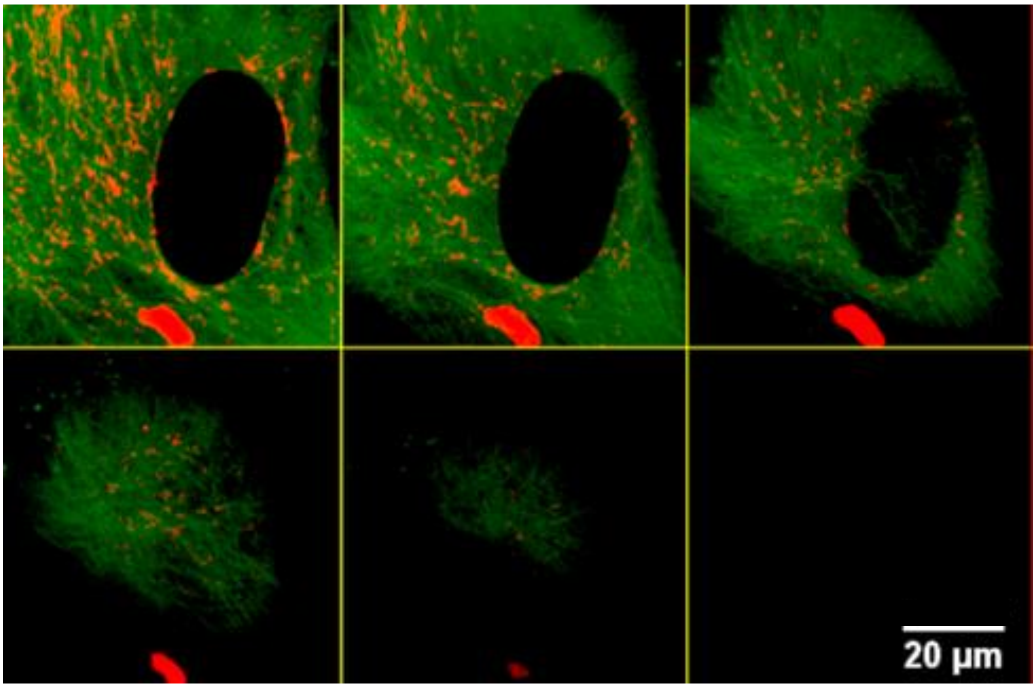

Supplement Figure S11-2. Retention of GNPs in CAFs. Z-stack showing the distribution of NPs across different planes starting from the adherent plane onwards. MTs and GNPs are labelled in green and red, respectively.

Supplementary section S12:

The relative growth (y) is described by

$$y = \frac{A}{\left\{1 + \exp\left[\frac{4\mu}{A}(\lambda - t) + 2\right]\right\}} \quad (1)$$

Where μ is the growth rate, A is the asymptote or maximum growth A, and λ is the lag time.

The values of fitted parameters such as μ and A from growth curves were used to calculate cell doubling time (T_d) as outlined in equation 2:

$$T_d = \ln(2) \cdot \left(\frac{4\mu}{A}\right)^{-1} \quad (2)$$

The calculated values of T_d for HeLa, NFs, and CAFs were19.5, 49.7, and77.0 hrs, respectively.

Finally, the survival fraction (SF) was calculated using the following formula:

$$SF = 2^{-\frac{T_{delay}}{T_d}} \quad (3)$$

Where T_d is the doubling time and T_{delay} is the delay time. T_{delay} is define as the time takes to reach the same growth in the treated curves compared to control after one doubling time.[34]

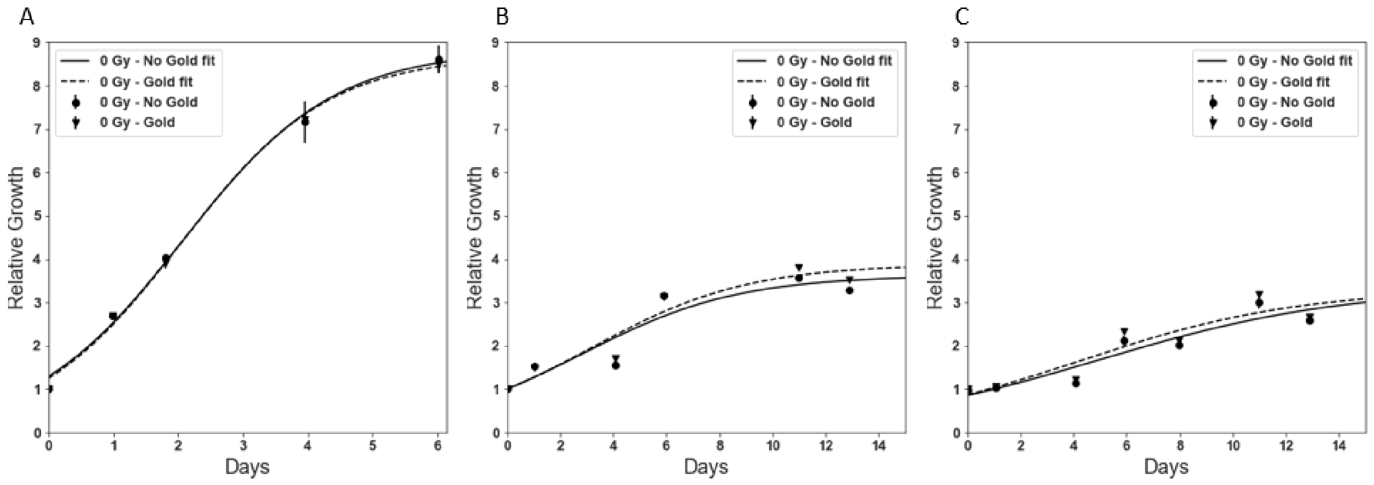

Supplement: Supplementary file 1 — Additional file 1: Supplementary Figures. [file 12645_2020_64_MOESM1_ESM.pdf]
